# Photoelectrochemical performance of BiOI/TiO_2_ nanotube arrays (TNAs) p-n heterojunction synthesized by SILAR-ultrasonication-assisted methods

**DOI:** 10.1098/rsos.221563

**Published:** 2023-06-28

**Authors:** Sherly Kasuma Warda Ningsih, Rahmat Wibowo, Jarnuzi Gunlazuardi

**Affiliations:** ^1^ Department of Chemistry, Faculty of Mathematics and Natural Sciences (FMIPA), Universitas Indonesia, Depok 16424, Indonesia; ^2^ Department of Chemistry, Faculty of Mathematics and Natural Sciences (FMIPA), Universitas Negeri Padang, Kampus Air tawar, Padang 25130, Indonesia

**Keywords:** BiOI, TiO_2_ nanotube, SILAR-ultrasonication, nanoplate, photocurrent

## Abstract

In order to extend the visible region activity of titania nanotube array (TNAs) films, the successive ionic layer adsorption and reaction (SILAR)-ultrasonication-assisted method has been used to prepare BiOI-modified TiO_2_ nanotube arrays (BiOI/TNAs). The band gap of BiOI/TNAs for all the variations reveals absorption in the visible absorption. The surface morphology of BiOI/TNAs is shown in the nanoplate, nanoflake and nanosheet forms with a vertical orientation perpendicular to TiO_2_. The crystalline structure of BiOI did not change the structure of the anatase TNAs, with the band gap energy of the BiOI/TNAs semiconductor in the visible region. The photocurrent density of the BiOI/TNAs extends to the visible-light range. BiOI/TNAs prepared with 1 mM Bi and 1 mM KI on TNAs 40 V 1 h, 50 V 30 min show the optimum photocurrent density. A tandem dye-sensitized solar cell (DSSC)-photoelectrochemical (PEC) was used for hydrogen production in salty water. BiOI/TNAs optimum was used as the photoanode of the PEC cell. The solar to hydrogen conversion efficiency (STH) of tandem DSSC-PEC reaches 1.34% in salty water.

## 1. Introduction

TiO_2_ has various morphologies, such as nanorods, film, nanoparticles and nanotubes [1]. TiO_2_ nanotube arrays (TNAs) have been used as photoelectrode. The extensive application of TiO_2_ is due to low cost, higher chemical stability [2] and non-toxicity [3] and high photocatalytic activity [4]. The recombination between the electron (e) and hole (h^+^) can be reduced by open channel 1D to transport charge [5–8].

The application of TNAs semiconductors is limited because their absorption is only in the ultraviolet (UV) region, fast photo-induced electron–hole pair recombination rate and low electrical mobility [3]. Due to this limitation, the absorption of TNAs needs to extend to the visible region by modifying the TNAs by doping metal ions [9] and non-metals [10,11]. Furthermore, the formation of heterojunctions can also resolve the weakness of TNAs. The heterojunction design can improved the transfer and separation efficiency [12]. The strategy to improve TNAs that can be changed into visible regions was the formation of a junction between n-type TNAs with p-type semiconductors with a narrow band gap [13,14], so it can work under visible light irradiation. The internal electric field can build by n-p heterojunction formation from the n-type direction to the p-type direction, which transfers electrons and holes.

The photocatalytic activities of TNAs can be improved by the combination of TNAs with another semiconductor to form a composite, such as BiVO_4_ [15], CdS [16], MoS_2_ [13], ZnIn_2_S_4_ [4] and Cu_2_O [14]. Bismuth oxyhalide (BiOX), including Cl, Br and I), has a structure with a layered structure. The layered structure is considered a promising photocatalyst [17,18]. Among BiOX semiconductors, BiOI is the semiconductor in p-type that has a narrowest band gap of *ca* 1.7 eV [19]. The photogenerated electron–hole recombination rate of pure BiOI is fast, so it results in low photocatalytic activity. Until now, there have been many reports about synthesizing BiOI/TNAs and their application. Li *et al*. conducted BiOI/TiO_2_ heterojunction through a simple hydrothermal process [20]. Dai *et al*. fabricated the BiOI/TNAs using an impregnated process and showed that the photoelectrocatalytic (PEC) application of BiOI/TNAs is higher than pure BiOI [21]. Liu *et al*. synthesized BiOI/TNAs using the SILAR method [22].

Hydrogen evolution by using semiconductor photocatalysts via solar water splitting is a promising strategy to solve the energy crisis [12]. The water splitting performed by a single PEC cell of BiOI/TNAs needs external potential or bias voltage to oxidize water. The visible light harvesting can be improved using tandem dye-sensitized solar cell (DSSC) and PEC cells with dyes photosensitizer for DSSC cells [23]. BiOI was modified on TNAs using SILAR-assisted ultrasonication methods on various TNAs matrices in this research. The morphology of BiOI deposited on TNAs shows nanoplate, nanoflake and nanosheet. The optimum photoelectrochemical performance of BiOI/TNAs was the BiOI/TNAs with 1 mM of cationic and anionic precursors concentration on TNAs for nine cycles of SILAR. BiOI/TNAs optimum was used as a photoanode for salty water splitting using a tandem DSSC-PEC system.

## 2. Material and methods

### 2.1. Materials

The chemical used in this research were titanium (Ti) foil (from Baoji Jhinseng Metal Material Co), NH_4_F, C_2_H_6_O_2_, Bi(NO_3_)_3_.5H_2_O, KI, HNO_3_ 0.4 M, ethanol, acetone, sodium sulfate, fluorine-doped tin oxide (FTO) glass, KI, I_2_, NaCl, H_2_PtCl_6_, N719 dyes, Nafion membrane and deionized water (DI).

### 2.2. Fabrication of BiOI/TNAs by SILAR-ultrasonication assisted method

**Figure 1.**
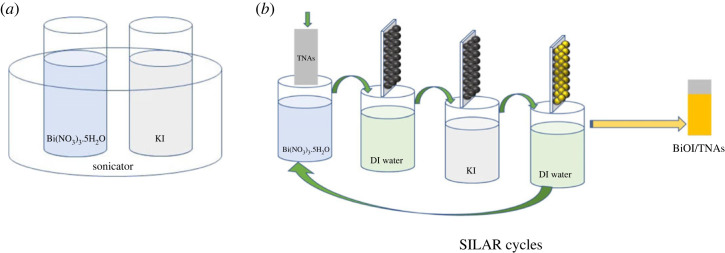
Illustration of SILAR-ultrasonication-assisted process of BiOI/TNAs.

TNAs were synthesized by modifying the second-step potentials of the two-step anodization method. The applied potential used in the first step method was fixed at 40 V for 60 min, and the second step anodization voltage was 50 V with various anodization times, i.e. 15, 30 and 60 min. BiOI/TNAs were prepared through the SILAR-ultrasonication-assisted method. The cationic precursor Bi(NO_3_)_3_.5H_2_O was used with various concentrations, and a solution of KI with various molar ratios was used as the anionic precursor. The deionized water was used as the solvent to dilute the precursors. TNAs foil was immersed in the cationic precursors with a time variation of 10, 45 and 90 s, followed by immersion in deionized water. After that, the TNAs foils were immersed in anionic precursors solution and then deionized water immersion to remove the not-adsorbed ion. SILAR cycles, the concentration of the precursors, and immersion time were varied to get the optimum condition. BiOI/TNAs were dried in open-air conditions. SILAR-ultrasonication-assisted process is shown in figure 1. For comparison, BiOI/TNAs also prepared by SILAR without ultrasonication assisted, that symbolized by S (SILAR without ultrasonication assisted).

### 2.3. Characterization of BiOI/TNAs

The BiOI/TNAs surface morphology and cross-section were analysed by scanning electron microscopy (SEM, FEI, Quanta 650). The selected area electron diffraction (SAED) and high resolution of the micrograph were characterized by transmission electron microscopy (TEM, FEI Tecnai G2 20 S-Twin). The optical structure of BiOI/TNAs was analysed by UV-visible diffuse reflection spectra (UV-Vis, 2450 Shimadzu). The crystalline structures of BiOI/TNAs were performed by X-ray diffractometer (XRD, XPERT PRO PANalytical). The Raman shift of BiOI/TNAs was characterized by Raman spectroscopy (HORIBA HR Evolution), and the photoluminescence was studied using photoluminescence (Horiba MicOS Photoluminescence).

### 2.4. Photoelectrochemical performances and hydrogen evolution of BiOI/TNAs in salty water

The photocurrent density of BiOI/TNAs heterojunctions was analysed by potentiostat with three electrode systems. The working electrode for the measurement used a sample of BiOI/TNAs with various concentrations. The Pt wire was used as the counter electrode, with the distance between Pt wires and the working electrode being 15 mm. Meanwhile, the reference electrode used Ag/AgCl (3 M KCl) electrode without applied bias potential. NaSO_4_ 0.1 M solutions were applied as the electrolyte in linear sweep voltammetry (LSV) technique and multiple pulse amperometry (MPA) analysis. The scan rate of LSV analysis was 25 mV s^−1^ in the visible illumination with a 13 W visible lamp. The MPA technique was performed with chopped dark and light illumination every 10 s without bias potential. The hydrogen evolution was performed using BiOI/TNAs photoanode with the optimum photocurrent density. A tandem system of DSSC-PEC cells was used with DSSC cells arranged by sandwich configuration. The DSSC cells comprised N719 dyes/TNAs on 40 V 1 h, 50 V 30 min as the anode, Pt decorated on FTO glass was used as the cathode. I^−^/I_3_^−^ electrolyte was used as the electrolyte [24]. The N719/TNAs anode was prepared by immersing TNAs in 0.3 mM N719 dyes in ethanol solvent at room temperature for 24 h with slight modification [25]. The electrolyte solution was prepared by dissolving 0.13 g of I_2_ crystal in 20 ml of acetonitrile and 5 ml of ethylene glycol. After that, 0.18 g of KI was added to this solution and mixed for 30 min [24]. The Pt/FTO cathode was prepared by dropping 40 mM H_2_PtCl_6_ in ethanol and then by a calcination process at 380°C [26]. The H-reactor compartment was used for the hydrogen evolution and connected by the Nafion membrane. NaCl 0.5 M solution and a 150 W metal halide lamp were used as the electrolyte and visible light sources. The hydrogen gas was analysed using gas chromatography (GC) with an activated carbon column.

## 3. Results and discussion

TNAs with various pore diameters were applied to prepare a p-n heterojunction with BiOI material with the SILAR-ultrasonication-assisted method. SILAR is the adsorption technique and ionic layer reaction with the electrical double-layer principles [27]. The reaction for preparation of BiOI/TNAs by the SILAR method is shown in equation (3.1).

3.1 $$\mathrm{Bi}{\left(\mathrm{OH}\right)}_{3}+I^{-}\to \mathrm{BiOI}+2H_{2}O+{\mathrm{OH}}^{-}.$$

### 3.1. Characterization of BiOI/TNAs heterojunction by SEM-EDX

**Figure 2.**
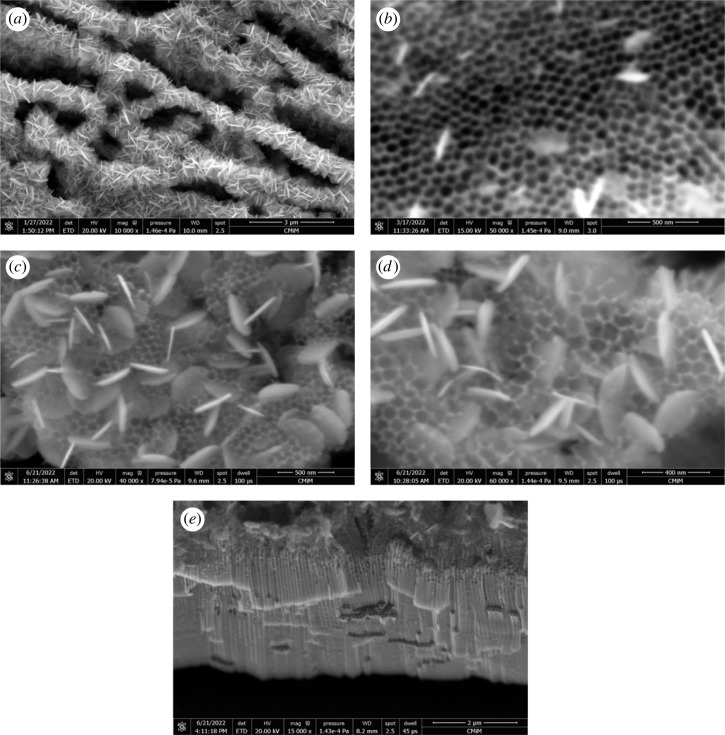
Surface morphology of BiOI/TNAs prepared of (*a*) 7 mM of cationic precursor and 7 mM of anionic precursor for 90 s with seven cycles (US1), (*b*) 2 mM of cationic precursor and 2 mM of anionic precursor for 45 s with five cycles (US6), (*c*) 1 mM of cationic precursor and 1 mM of anionic precursor for 45 s with nine cycles (US23), (*d*) 1 mM of cationic precursor and 1 mM of anionic precursor for 10 s with 17 cycles (US25) and (*e*) cross-section image of BiOI/TNAs prepared of 1 mM of cationic precursor and 1 mM of anionic precursor for 10 s with 17 cycles (US25) (TNAs 40 V 1 h, 50 V 60 min).

Figure 2 exhibits the surface morphology of BiOI/TNAs fabricated on TNAs (TNAs 40 V for 60 min in the first step and 50 V for 60 min in the second step). The concentration of cationic and anionic precursors was 7 mM (figure 2*a*), with the cycles of SILAR that were applied to be seven time cycles (US1). The SEM images of the sample of BiOI were in the nanoflake with self-assembly formed garland flower-like. The garland flower-like morphology of BiOI is due to the intrinsic layer structure of tetragonal BiOI. The morphology of TNAs was hexagonally structured (honeycomb) with a diameter pore of 75.97 ± 10.78 nm and a wall thickness of 11.04 ± 2.24 nm. The thickness and width of the sheet or flake of BiOI were 27.33 and 442.90 nm, respectively. The prepared BiOI morphology was very interesting, with the square form with an edge angle of approximately 90°. This nanosheet was formed in the tetragonal BiOI and crystallized to form a nanosheet with a thin thickness along the axis zone (101) [28]. BiOI flake was formed vertically on TNAs. SILAR growth of BiOI on TNAs performed at room temperature and atmospheric conditions.

The nanoflake shape will influence the contact area between the electrode and electrolyte solution. This shape will be profitable for electrolyte diffusion into the cavity of BiOI [29]. The overloading of BiOI will cause BiOI with the thicker layer, and the mouth of tube TNAs blocked with BiOI at 7 mM of cationic and anionic precursors. The overloading of BiOI on TNAs inhibited the light of injection, inhibiting light absorption and reducing the photo-induced electron–hole generation.

Figure 2*b* shows the morphology surface of BiOI/TNAs after SILAR was applied with the concentration of precursors of 2 mM (US6). The width and length of the sheet of BiOI were 26.67 and 176.08 nm, respectively. The cycles of SILAR were five times. The BiOI sheet's size decreased at this concentration variation compared with 7 mM precursor concentration (figure 2*a*). The morphology of BiOI at 2 mM of precursor concentration did not block the mouth of tube TNAs or the surface of TNAs. In these images, it also can be seen that the BiOI deposited at the wall tube of TNAs (figure 2*b*). From these results, the decreasing concentration can reduce the size of BiOI sheet. SEM images of BiOI/TNAs at precursor concentration were 1 mM of Bi(NO_3_)_3_.5H_2_O and 1 mM of KI for 45 s immersion process with nine cycles (US23) shown in figure 2*c*. BiOI morphology reveals that the nanodisc formed with a thickness of approximately 33.76 nm. SEM images of BiOI/TNAs at 17 cycles (US 25) were 36.49 nm (figure 2*d*). The BiOI sheet is deposited on the surface, and some sheets are implanted in the internal tube of TNAs. Figure 2*e* shows the cross-section of BiOI/TNAs for 17 cycles (US25). The EDX analyses of BiOI/TNAs prepared with 7 mM of cationic and anionic precursor show Ti, O, Bi and I atomics with composition was 35.16; 48.45; 8.46; and 7.93 At%, respectively (electronic supplementary material, figure S1 and table S1).

**Figure 3.**
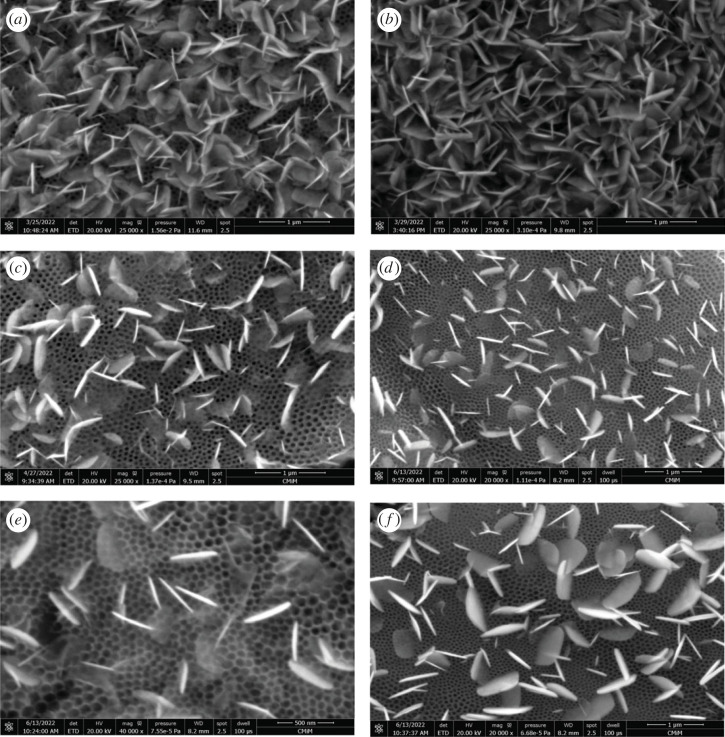
SEM micrograph of BiOI/TNAs prepared of (*a*) 5 mM of cationic precursor and 3 mM of anionic precursor for 45 s with five cycles (US9), (*b*) 5 mM of cationic precursor and 3 mM of anionic precursor for 45 s with five cycles (TNAs 40 V 45 min, 50 V 15 min) (US10), (*c*) 3 mM of cationic precursor and 2 mM of anionic precursor for 45 s with seven cycles (US12), (*d*) 1 mM of cationic precursor and 1 mM of anionic precursor for 45 s with seven cycles (US20), (*e*) 1 mM of cationic precursor and 1 mM of anionic precursor for 45 s with nine cycles (US21) and (*f*) 0.5 mM of cationic precursor and 0.5 mM of anionic precursor for 45 s with 20 cycles (US19) (TNAs 40 V 1 h, 50 V 15 min).

SEM micrographs of the BiOI/TNAs at condition anodization of TNAs of 40 V for 60 min at the first step and 50 V for 15 min at the second step are depicted in figure 3*a*. The precursor concentration of cationic and anionic BiOI were 5 and 3 mM for five time cycles, respectively (US9). From these images, the BiOI in nanosheet form is evenly distributed at the surface of TNAs. This nanosheet is assembled to produce nanoflower. The size and width of the sheet were 42.23 and 380.12 nm, respectively.

Figure 3*b* shows the SEM images of BiOI deposited on TNAs (40 V 45 min for the first step and 50 V 15 min for the second step) with the concentration of the cationic and anionic precursors were 5 mM and 3 mM, respectively, as shown in figure 3*b*. The cycles of SILAR for this variation were seven cycles (US10). Figure 3*c* shows the SEM images of BiOI/TNAs that prepared with 3 mM of cationic precursor and 2 mM of anionic precursor for 45 s with seven cycles (US12). The SEM images show the nanosheet form on the surface of TNAs perpendicular to the TNA matrix. The width and length of the sheet were 26.34 and 306.73 nm, respectively. The SEM surface images and cross-section of BiOI/TNAs at precursor concentration 1 mM and seven cycles for 45 s (US20) can be seen in figure 3*d*. The morphology of BiOI/TNAs was in sheet form, and the size of width and the length of the sheet were 34.96 and 306.76 nm. The tube length of TNAs was 2.59 µm. In this cross-section can be seen there is the sheet of BiOI. The sheet of BiOI is not only at the surface of TNAs but also in this cross-section of TNAs. SEM images and cross-section of BiOI/TNAs at 1 mM of precursor concentration for nine SILAR cycles (US21) can be seen in figure 3*e*. The BiOI sheet detected at the surface of TNAs was more uniform, and the length and width of the sheet were 35.59 and 325.69 nm, respectively. The SEM surface morphology and the cross-section of BiOI/TNAs at a concentration of precursor of 0.5 mM (US19) can be seen in figure 3*f*. The morphology of BiOI was nanoplate form with the size of the thickness and the length of the plate was 53.65 and 517.34 nm, respectively, for 20 times of SILAR cycles. As shown from SEM images, the BiOI flake or sheet was also implanted into the inner tube of TNAs. The EDX mapping of BiOI/TNAs prepared with 5 mM of cationic precursor and 3 mM of the anionic precursor for 45 s with five cycles show the composition of Ti, O, Bi and I atoms was 52.5, 34.1, 9.9 and 3.5 wt%, respectively (electronic supplementary material, figures S2 and S3).

**Figure 4.**
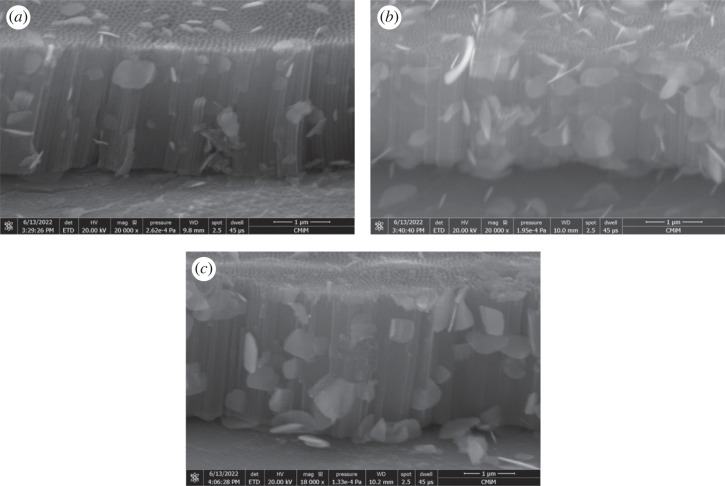
Cross-section images of BiOI/TNAs prepared of (*a*) 1 mM of cationic precursor and 1 mM of anionic precursor for 45 s with seven cycles (US20), (*b*) 1 mM of cationic precursor and 1 mM of anionic precursor for 45 s with nine cycles (US21) and (*c*) 0.5 mM of cationic precursor and 0.5 mM of anionic precursor for 45 s with 20 cycles (US19) (TNAs 40 V 1 h, 50 V 15 min).

Figure 4 shows the cross-section of BiOI/TNAs prepared with 1 mM and 0.5 mM precursors concentration. From figure 4*a*, the tube length was 2.59 µm. In this cross-section can be seen there is the sheet of BiOI. The SEM images proved that the sheet of BiOI is not only at the surface of TNAs but also in this cross-section. The cross-section of BiOI/TNAs for nine times of SILAR cycles is shown in figure 4*b*. The tube length was 3 µm, and in this cross-section exists the sheet of BiOI. Figure 4*c* shows the cross-section of BiOI/TNAs at 0.5 mM concentration of precursors for 20 times SILAR cycles.

**Figure 5.**
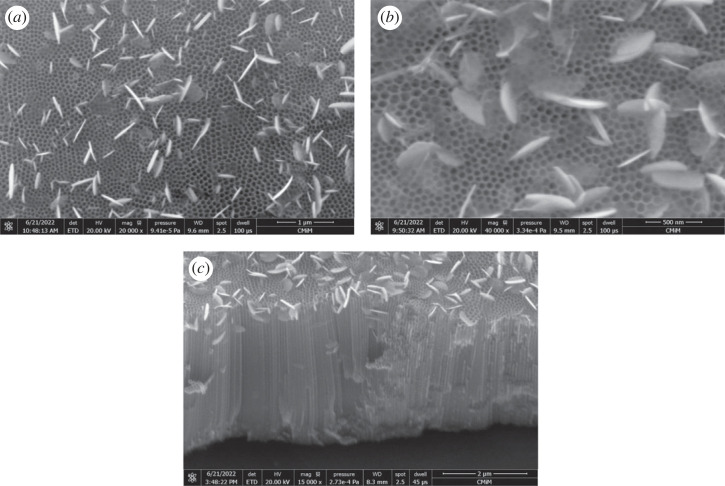
SEM images of BiOI/TNAs prepared of (*a*) 9-BiOI/TNAs US 45 s with 1 mM of cationic precursor and 1 mM of anionic precursor for 45 s with nine cycles (US22), (*b*) for 10 s, 17 cycles (US24) and cross-section of BiOI/TNAs (TNAs 40 V 1 h, 50 V 30 min).

The SEM images and the cross-section of BiOI/TNAs at precursors concentration of 1 mM for TNAs (anodized at 40 V for 1 h in the first step and 50 V for 30 min in the second step) can be seen in figure 5. The morphology of BiOI/TNAs for nine times SILAR cycles (US22) was in nanoflake. The width and length of the flake were 53.23 and 179.62 nm, respectively. The BiOI that was deposited on the TNAs matrix was perpendicular to the matrix, and some flakes of BiOI were implanted into the inner tube of TNAs. BiOI/TNAs for 10 s with 17 times SILAR cycles (US24) are shown in figure 5*b*. The thickness of the sheet was 37.42 nm. Figure 5*c* shows the cross-section of BiOI/TNAs at 10 s with 17 times SILAR cycles. From this cross-section can be seen the BiOI sheet that is sticking not much compared with 45 s of SILAR times. The EDX analysis of BiOI/TNAs that were prepared with 1 mM of cationic and anionic precursor for 45 s with nine cycles shows Ti, O, Bi and I atomic was 30.53, 68.40, 0.68, and 0.39 At% (electronic supplementary material, figure S4 and table S2). The BiOI/TNAs morphology has resulted in flake or sheet form. The internal electric field is perpendicular to the substrate, which is helpful for electron and hole photogeneration [30]. The increasing SILAR cycles can increase the photocurrent density. However, too many SILAR cycles reduce the photocurrent density due to dense distribution, disturbing the mass interface [30]. The presence of this BiOI nanoflake morphology will harvest light with higher efficiency. The photocurrent density of BiOI/TNAs will reduce with more BiOI deposited on TNAs; this is related to the reflection of visible light on the surface of TNAs above the coaxial BiOI/TNAs, and this is also confirmed from the results [31]. The essential photocatalyst activity is the uniform size and morphology of BiOI/TNAs with controllable [32].

### 3.2. Characterization of BiOI/TNAs heterojunction by HRTEM

**Figure 6.**
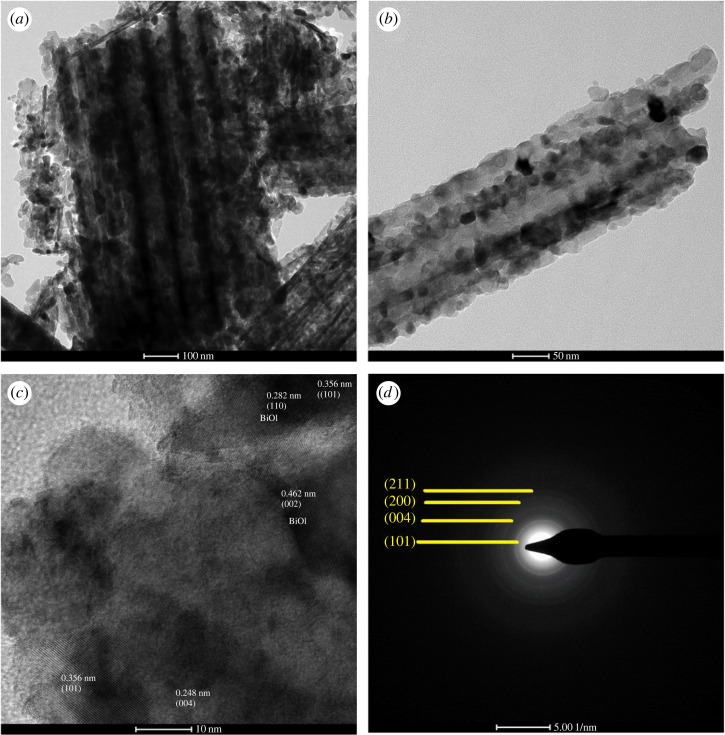
(*a*) and (*b*) TEM micrographs, (*c*) HRTEM images and (*d*) SAED of BiOI on TNAs 40 V 1 h in the first step and 50 V for 1 h in the second step anodization.

Figure 6 shows the TEM, HRTEM and SAED of BiOI/TNAs that were synthesized on TNAs (40 V 1 h in the first step and 50 V 1 h in the second step) at 1 mM of cationic and anionic precursors. The SILAR times were 45 s for 10 times of SILAR cycles. Figure 6*a,b* shows TEM images of BiOI/TNAs with tube morphology of TNAs and BiOI deposited on the surface of TNAs in nanoflake form. From figure 6*c*, the HRTEM of BiOI/TNAs with the fringes spacing of TNAs was 0.356 (101 lattice spacing) for the anatase phase of TiO_2_ (JCPDS no. 00-21-1272). The HRTEM image also detected the fringes lattice of BiOI with interplanar distances of 0.282 nm (101) and indicated that the tetragonal BiOI is matched with JCPDS no. 73-2062. These results showed that the prepared BiOI/TNAs with p-n junction consists of BiOI and TNAs in the clear crystal phase [33]. This observation is in accordance with the XRD result and the SEM micrograph.

### 3.3. Characterization of BiOI/TNAs heterojunction by XRD

**Figure 7.**
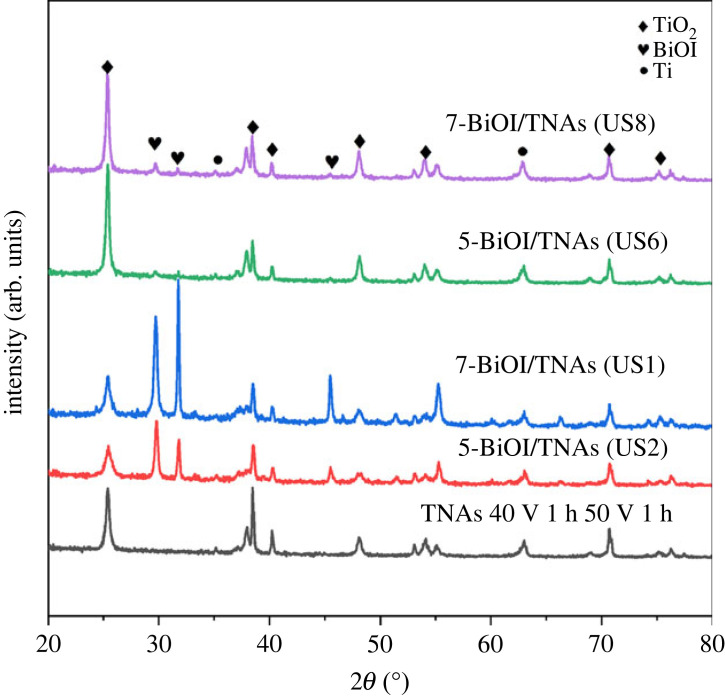
Diffractograms of BiOI/TNAs prepared on TNAs (40 V for 1 h in the first step and 50 V for 1 h in the second step).

Figure 7 shows a diffractogram of BiOI/TNAs prepared on TNAs (40 V for 1 h in the first step and 50 V for 1 h in the second step) with variations in concentration precursors and SILAR cycles. This XRD pattern showed that TiO_2_ peaks at 2*θ*: 25.40°, 38.54°, 48.10°, 54.13°, 55.25°, 70.74° and 76.35°, with Miller index of (101), (004), (200), (105), (211), (220) and (301), respectively. These seven peaks correspond to the standard of JCPDS no. 00-021-1272 with the anatase phase. Besides the anatase peaks of TNAs there are also BiOI peaks at 2*θ*: 29.79°, 31.78° and 45.53° with Miller Index of (012), (110) and (200), respectively. The structure of BiOI is the tetragonal structure that is according to the standard of JCPDS no. 73-2062 or ICDD no. 01-075-5209 [34]. On this diffractogram, there are no impurities such as BiI_3_, Bi_2_O_3_ and Bi_5_O_7_I. XRD peaks data with facets of (012) and (110) are confirmed with SEM image results, in which BiOI is perpendicular to the TNAs substrate. BiOI/TNAs with the highest peaks of facet (110) will be helpful in photocatalytic performance. The Ti peaks can be seen at 2*θ*: 35.21° and 61.69° with Miller index (100) and (213) (JCPDS no. 21-1294 or ICDD no. 01-089-4893). The modification of TNAs with BiOI showed no change in the anatase peaks of TNAs [35]. These diffractograms show no impurities peaks, such as the Bi atom and the I atom, in BiOI samples.

The diffraction peaks of BiOI synthesized using SILAR ultrasonication assisted were higher than without ultrasonication assisted (7-BiOI/TNAs S 60 s 7 mM of cationic and anionic precursor. The XRD results also confirmed the SEM images of 7 mM of cationic and anionic precursors with a higher atomic composition of Bi atom and I atom than without ultrasonication assistance. The BiOI diffraction peaks show increasing BiOI composition in the sample. A diffractogram of BiOI/TNAs synthesized at precursor concentration 2 mM of Bi(NO_3_)_3_.5H_2_O and 1 mM KI (ratio 2 : 1) is shown in figure 7. The BiOI/TNAs were prepared with SILAR cycles of five, seven and nine times cycles. There are three groups of peaks on the diffractograms, such as TiO_2_, Ti and BiOI peaks. The anatase peaks of TNAs were detected at 2*θ*: 25.4°, 38.5°, 48.2°, 54.0°, 55.3°, 70.8° and 76.4°, with Miller index of (101), (004), (200), (105), (211), (220) and (301), respectively. These peaks are fixed with JCPDS no. 00-021-1272 with the anatase phase. The BiOI peaks were detected at 2*θ*: 29.8°, 31.8° and 45.6°, with Miller index (012), (110) and (200) with the tetragonal structure of BiOI (JCPDS no. 73-2062). The Ti peaks can be observed at 2*θ*: 35, 2° and 62° with Miller index of (100) and (213) (JCPDS no. 21-1294). The diffraction peak of BiOI/TNAs prepared at five, seven and nine times cycles show the anatase peak intensity higher than BiOi peaks. The FWHM for the 7-BiOI/TNAs and 9-BiOI/TNAs are narrow and high intensity. The SILAR cycles of BiOI are proportional to the BiOI deposited at TNAs.

**Figure 8.**
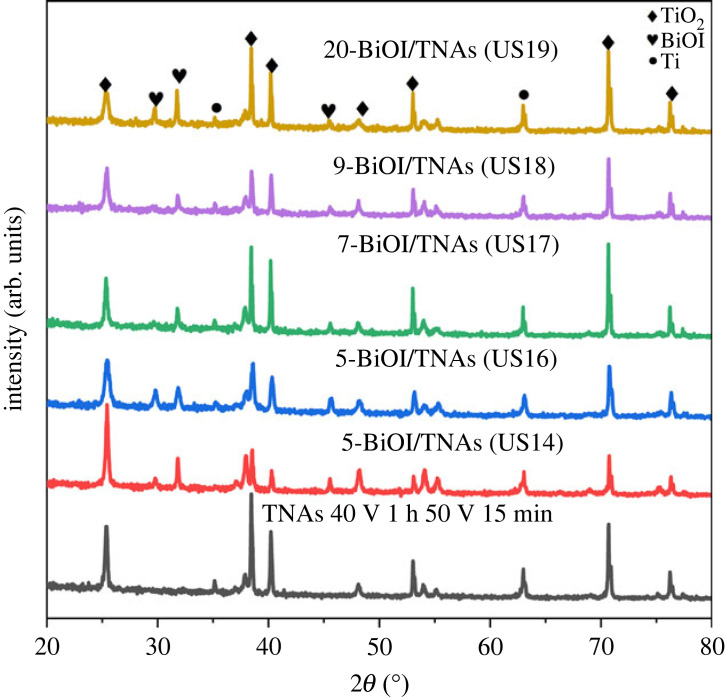
Diffractograms of BiOI/TNAs prepared on TNAs (40 V for 1 h in the first step and 50 V for 15 min in the second step).

The diffractograms of BiOI/TNAs prepared (40 V for 1 h in the first step and 50 V for 15 min in the second step) with a precursor concentration of 0.5 mM are shown in figure 8. From this diffractogram, the three peaks of TiO_2_, Ti and BiOI. The anatase peaks of TNAs exist at 2*θ*: 25.4°, 38.5°, 48.2°, 54.0°, 55.3°, 70.8° and 76.4° with Miller Index of (101), (004), (200), (105), (211), (220) and (301) (JCPDS no. 021-1272). Whereas, BiOI peaks can be observed at 2*θ*: 29.8°, 31.8° and 45.6°, with Miller index of (012), (110) and (200) with tetragonal structure (JCPDS no. 73-2062). BiOI/TNAs peaks for 20 times of SILAR cycles with 0.5 mM concentration (1:1 ratio) can be seen that the BiOI intensity is high and FWHM is narrow.

**Figure 9.**
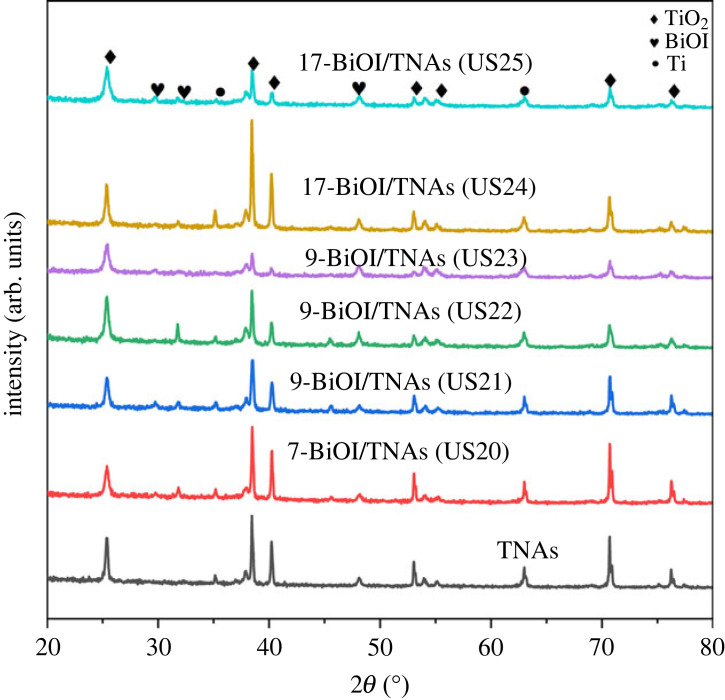
Diffractograms of TNAs and BiOI/TNAs prepared with a concentration of 1 mM of cationic precursor and 1 mM of anionic precursor on TNAs (40 V for 1 h in the first step and 50 V for 15 min (US20 and (US21)), (40 V for 1 h min in the first step and 50 V for 30 min in the second step (US22 and US24)), and TNAs C (40 V for 1 h min in the first step and 50 V for 1 h in the second step (US23 and US25)).

XRD characterization of BiOI/TNAs prepared on TNAs (40 V for 1 h in the first step and 50 V for 30 min, and 50 V for 60 min in the second step) with precursors concentration of 1 mM can be seen in figure 9. SILAR duration per cycle was 45 s for nine times and 10 s for 17 cycles. The diffractogram has three peaks: TiO_2_ anatase, tetragonal BiOI and Ti. Anatase peaks are detected at 2*θ*: 25.38°, 38.49°, 48.09°, 54.07°, 55.10°, 70.74° and 76.4°, with Miller index of (101), (004), (200), (105), (211), (220) and (301), respectively (JCPDS no. 00-021-1272). BiOI peaks are detected at 2*θ*: 29.8°, 31.77° and 45.51°, with Miller index (012), (110) and (200) with tetragonal structure (JCPDS no. 73-2062). Ti peaks exist at 2*θ*: 35.14° and 63.01° with Miller index of (100) and (213) (JCPDS no. 21-1294). The intensity of 9-BiOI/TNAs B (45 s) is higher than that of BiOI/TNAs C (45 s). In comparison, 17-BiOI/TNAs C (10 s) is higher than 17-BiOI/TNAs B (10 s).

### 3.4. Characterization of BiOI/TNAs heterojunction by UV-Vis DRS

**Figure 10.**
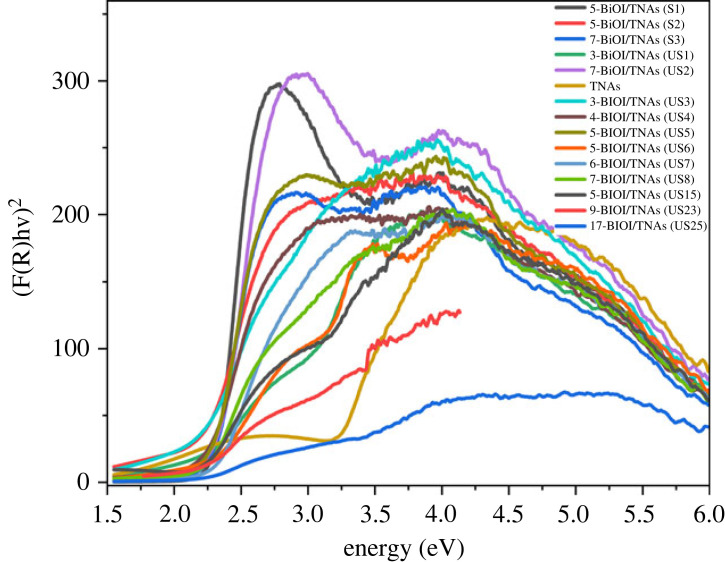
Tauc plots of BiOI/TNAs 40 V 1 h, 50 V 1 h.

Figure 10 depicts the Tauc plots of BiOI/TNAs on TNAs 40 V 60 min 50 V 60 min. These spectra show that TNAs only absorbed the UV light lower than 400 nm, i.e. 380 nm [29]. Kulbeka–Munk equation can calculate the band gap energy and Tauc plot approximately 3.1 eV, showing the anatase phase [36] and [29]. Pristine BiOI has a wavelength absorption at 670 nm [37] with a band gap energy of 1.86 eV [36]. Pristine BiOI has a weakness, namely the fast recombination of electrons and holes [38]. BiOI has been deposited on TNAs with 7 mM precursor concentration by SILAR method without ultrasonication as a comparison by varying the SILAR cycles. The variation of SILAR was five times for 45 s (S1), five times for 60 s (S2) and seven times for 60 s per cycle (S3). The optical response of BiOI/TNAs at all variations shows the shift to visible light in the 400–650 nm range. The band gap energy for this variation was 2.17, 2.19 and 2.16 eV, respectively. This band gap calculation showed the increase of SILAR cycles, so absorption to visible light and redshift.

The BiOI/TNAs prepared at 7 mM precursors concentration using SILAR with ultrasonication assisted also show the absorption in visible light. Band gap energy for BiOI deposited at TNAs for three times (US1), and seven times cycles (US2) were 2.14 and 2.10 eV, respectively. TNAs deposited with BiOI at all SILAR time variations showed a redshift in the visible wavelength of 400–650 nm [36] and [22]. BIOI/TNAs modified with SILAR-ultrasonication assisted show the narrower band gap energy compared with BiOI/TNAs by SILAR-without ultrasonication. The band gap energy of BiOI/TNAs at 7 mM precursors concentration can be seen in table 1.

**Table 1.** Band gap energy of BiOI/TNAs at various concentrations of cationic and anionic precursors.

synthesis conditions	sample code	the band gap energy (eV)
7 mM of cationic precursor and 7 mM of anionic precursor		
TNAs	S0	3.15
5-BiOI/TNAs S 45 s	S1	2.17
5-BiOI/TNAs S 60 s	S2	2.19
7-BiOI/TNAs S 60 s	S3	2.16
3-BiOI/TNAs US 90 s	US1	2.14
7-BiOI/TNAs US 90 s	US2	2.10
3 mM of cationic precursor and 2 mM of anionic precursor		
3-BiOI/TNAs US 60 s	US3	2.12
4-BiOI/TNAs US 60 s	US4	2.23
5-BiOI/TNAs US 60 s	US5	2.28
2 mM of cationic precursor and 2 mM of anionic precursor		
5-BiOI/TNAs US 45 s	US6	2.24
6-BiOI/TNAs US 45 s	US7	2.13
7-BiOI/TNAs US 45 s	US8	2.24
5 mM of cationic precursor and 3 mM of anionic precursor		
5-BiOI/TNAs US 45 s TNAs A	US9	2.17
5-BiOI/TNAs US 45 s TNAs D	US10	2.23
3 mM of cationic precursor and 2 mM of anionic precursor		
5-BiOI/TNAs US 45 s TNAs A	US11	2.29
7-BiOI/TNAs US 45 s TNAs A	US12	2.23
9-BiOI/TNAs US 45 s TNAs A	US13	2.08
3 mM of cationic precursor and 3 mM of anionic precursor		
5-BiOI/TNAs US 45 s TNAs A	US14	2.31
5-BiOI/TNAs US 45 s TNAs C	US15	2.16
2 mM of cationic precursor and 1 mM of anionic precursor		
5-BiOI/TNAs US 45 s TNAs A	US16	2.32
7-BiOI/TNAs US 45 s TNAs A	US17	1.91
9-BiOI/TNAs US 45 s TNAs A	US18	2.26
0.5 mM of cationic precursor and 0.5 mM of anionic precursor		
20-BiOI/TNAs US 45 s TNAs A	US19	1.90
1 mM of cationic precursor and 1 mM of anionic precursor		
7-BiOI/TNAs US 45 s TNAs A	US20	2.10
9-BiOI/TNAs US 45 s TNAs A	US21	1.92
9-BiOI/TNAs US 45 s TNAs B	US22	2.22
9-BiOI/TNAs US 45 s TNAs C	US23	1.97
17-BiOI/TNAs US 10 s TNAs B	US24	2.19
17-BiOI/TNAs US 10 s TNAs C	US25	2.21

**Figure 11.**
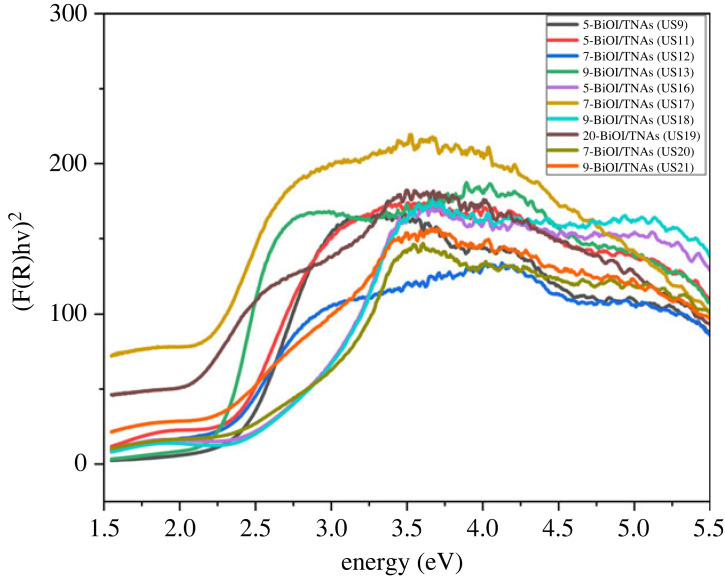
Tauc plots of BiOI/TNAs 40 V 1 h, 50 V 15 min.

The UV-Vis DRS spectra of BiOI/TNAs that were prepared at 5 mM of cationic precursor and 3 mM of anionic precursor on TNAs 40 V 1 60 min 50 V 15 min (US9) and 40 V 45 min 50 V 15 min (US10) shown in figure 11. The band gap energy of BiOI/TNAs of five times cycles on TNAs 40 V 60 min, 50 V 15 min (US9) and 40 V 45 min, 50 V 15 min (US10) was 2.17 and 2.23 eV. The spectra of UV-Vis DRS of BiOI/TNAs synthesized with concentration variation of 3 mM of cationic precursor and 2 mM of anionic precursor with five (US11), seven (US12) and nine times (US13) also can be seen in figure 11. The band gap energy of BiOI/TNAs at this condition was 2.29, 2.23 and 2.08 eV, respectively. The band gap energy of BiOI/TNAs with 3 mM of cationic precursor and 3 mM of anionic precursor on TNAs 40 V 1 h, 50 V 15 min (US14) and TNAs 40 V 60 min, 50 V 60 min is shown in figure 11. The band gap energy of this variation was 2.31. The UV-Vis DRS spectra of BiOI/TNAs at 2 mM of cationic precursor and 1 mM of anionic precursor can be seen in figure 11. The SILAR cycles were five (US16), seven (US17) and nine cycles (US18) with band gap energy of 2.32, 1.92 and 2.26 eV, respectively. For the precursor's concentration of 0.5 mM 20 times (US19), SILAR cycles are shown in figure 11. The band gap energy of BiOI/TNAs at the 20 times cycle was 1.90 eV. Meanwhile, 1 mM of precursor concentration for seven (US20) and nine SILAR cycles (US21) can be seen in figure 11, with the band gap energy being 2.10 and 1.92 eV, respectively. The band gap energy of 20 SILAR cycles in the nanoplate morphology (US19). Otherwise, the band gap energy is more extensive, producing nanoflake morphology. These results were also confirmed with the SEM images.

**Figure 12.**
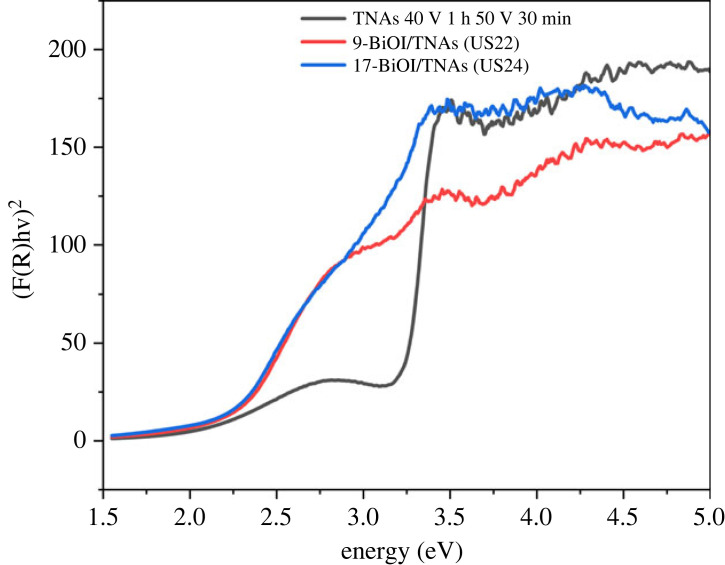
Tauc plots of BiOI/TNAs of 40 V 1 h, 50 V 30 min.

The UV-Vis DRS spectra of BiOI/TNAs synthesized with 1 mM of cationic precursor and 1 mM of anionic precursor on TNAs 40 V 60 min, 50 V 30 min is presented in figure 12. The SILAR cycle was five times for 45 s for each cycle (US22) and 17 times for 10 s per cycle (US24). The band gap energy of BiOI/TNAs for nine times was 2.22 eV. Otherwise, the band gap energy of BiOI/TNAs for 17 times SILAR cycles was 2.19 eV. Based on the band gap calculation, BiOI deposited on TNAs shows absorption to visible light in the range of 400–650 nm due to the intrinsic of BiOI. The BiOI material has a narrow band gap that absorbs the visible light region and belongs to a p-type semiconductor [21].

Based on the results of this band gap calculation, BiOI deposited on TNAs shows absorption in visible light in the range of 400–650 nm due to the intrinsic absorption band gap of BiOI. The BiOI is a p-type semiconductor with a narrow band gap [21], and TiO_2_ is an n-type semiconductor with a wide band gap value (3.2 eV) [39]. The heterojunction with a smaller band gap indicates a higher valence band position and a lower conduction band will increase the production of electrons and holes [40]. The position potential of the conduction band (CB) and the valence band edge of BiOI between pristine TNAs and BiOI were estimated according to the concept of electronegativity [35,41]. The geometric average electronegativity for each semiconductor (*χ*TiO_2_ and BiOI) can be calculated using equation (3.2).

3.2 $$\chi {\mathrm{TiO}}_{2}=\sqrt[{3}]{\chi \mathrm{Ti}\chi O^{2}}.$$

The valence and conduction band potentials of the two semiconductors at the point of zero charges can be calculated using equations such as (3.3) [42].

**Figure 13.**
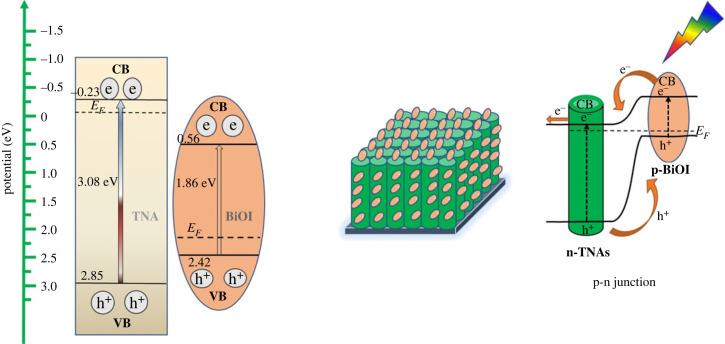
Schematic diagram of energy band structure n-TiO_2_ and p-BiOI.

3.3 $$E_{\mathrm{VB}}=\chi -E^{e}+0.5{\;E}_{g},$$

where *χ* is the absolute electronegativity of the semiconductor, which is defined as the geometric mean of the electronegativities of the constituent atoms. *E*^e^ is the energy of free electrons on the hydrogen scale (ca. 4.5 eV). *E*_VB_ is the edge potential of the valence band (VB), and *E*_CB_ is the position of the edge of CB with the formula *E*_CB_ = *E*_VB_−*E*_g_ [43]. The values of *χ*Ti and *χ*O are 3.45 and 7.54 eV, respectively. The value of *χ* TiO_2_ is 5.90 eV, and *χ*BiOI is 5.94 eV. The positions of CB and VB of BiOI and TNAs were calculated using equation (3.3) and are depicted in figure 13.

TNAs are n-type semiconductors with a Fermi level (EF) position close to their CB, and BiOI is a p-type semiconductor with a Fermi level (EF) close to their VB [35]. The E(.OH/OH^−^) and E(.OH/H_2_O) was +1.99 and +2.68 eV, respectively [4]. The preferred band structure of BiOI and TNAs is to form type II after contact to facilitate electron transfer from CB of BiOI to TNAs and hole transfer from VB of TNAs to BiOI. For Fermi equilibrium, there is a directional migration of electrons between the BiOI and TNAs, which shows a decrease in the energy band of the TNAs and an increase in the energy band of BiOI and the electric field in the p-n junction region. With exposure to visible light, both BiOI and TNAs can be excited. The difference in energy levels between TNAs and BiOI causes excited electrons and holes to flow, thereby reducing the recombination between electrons and holes. The presence of an internal electric field promotes the separation of electrons and holes. The electrons and holes that are formed due to this light will be transferred to the surface for the PEC reaction.

### 3.5. Characterization of Raman spectroscopy

**Figure 14.**
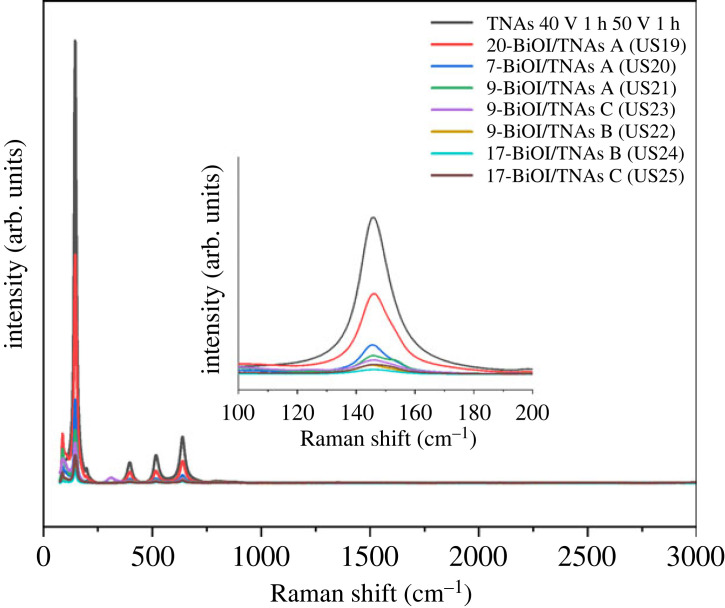
Raman spectra of BiOI modified on TNAs (40 V 1 h, 50 V 15 min/ TNAs A), 40 V 1 h, 50 V 30 min (TNAs B) and 40 V 1 h, 50 V 1 h (TNAs C).

The Raman spectra of BiOI/TNAs is shown in figure 14. From these spectra can be seen the Raman shift of TiO_2_ anatase, vibration stretching symmetry of O-Ti-O at 143.97 cm^−1^ (E_g_), vibration peaks of Ti-O at 396.8 cm^−1^ (B_1g_), 513.98 cm^−1^ (B_1g_) and 636.45 cm^−1^ (E_g_). This Raman shift is in accordance with TiO_2_ pristine [3,44]. These results are confirmed by XRD analysis. E_g_ and B_1g_ were the vibration stretching symmetry and vibration bending symmetry from O-Ti-O bonding, respectively [45]. BiOI deposited on TNAs shows the specific Raman peaks of TNAs and Raman shift detected. The sample of 20-BiOI/TNAs 0.5 mM of cationic precursor and 0.5 mM of anionic precursor have specific Raman peaks at 88.27 cm^−1^ that showed Bi-I mode and at 14 611 cm^−1^ (E_g1_) peak mode for vibration stretching symmetry of O-Ti-O, 397.10 cm^−1^ (B_1g_), 516.93 cm^−1^ (B_1g_) and 639.19 cm^−1^ (E_g1_). The mode E_g1_ intensity of bare TNAs is higher than that of E_g1_ of TNAs after being deposited with BiOI under several conditions. The mode of Bi-I peaks at 7-BiOI/TNAs (40 V for 60 min and 50 V for 15 min), 9-BiOI/TNAs (40 V 60 min, 50 V 15 min), 9-BiOI/TNAs (40 V 60 min, 50 V 60 min) exist at Raman shift of 88.27, 87.75, 88.27 and 88.27 cm^−1^, respectively. The BiOI pristine with a tetragonal structure has vibrational modes at 2A_1g_, B_1g_, 3E_g_, 2E_u_ and 2A_2u_ [46]. The vibrational mode at 2A_1g_, B_1g_ and 3E_g_ shows Raman mode active; otherwise, 2E_u_ and 2A_2u_ were IR active modes [47]. The increase in the vibrational peak of Bi-I indicates that a lot of BiOI is deposited on TNAs. The large molarity will induce agglomeration and aggregation of BiOI particles, increasing the amount of BiOI [48].

### 3.6. Characterization of BiOI/TNAs by photoluminescence

**Figure 15.**
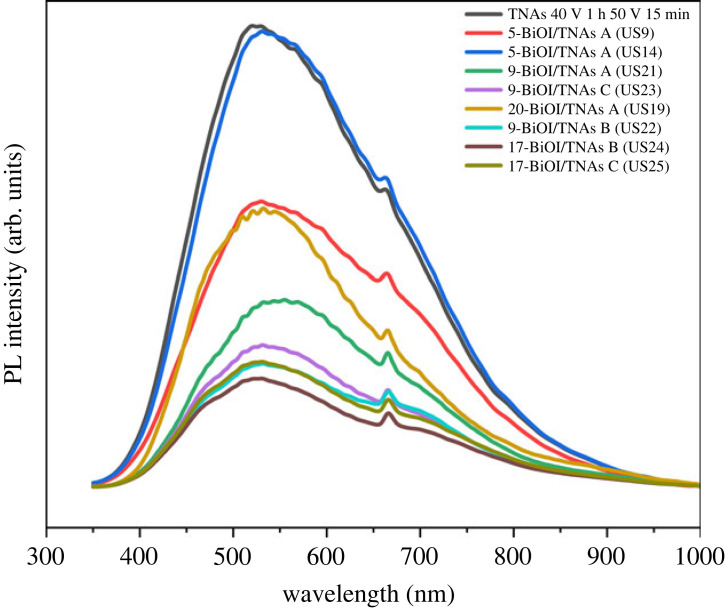
PL spectra of BiOI modified on TNAs: 40 V 1 h, 50 V 15 min (TNAs A), 40 V 1 h, 50 V 30 min (TNAs B) and 40 V 1 h, 50 V 1 h (TNAs C).

The charge separation of BiOI/TNAs was analysed using photoluminescence (PL). The highest separation efficiency of photoexcited charge carriers indicates the effective photocatalytic performance of photocatalysts [41]. In PL spectra the excitation wavelength 325 nm was used. The electrons in the ground state are excited to transition in the conduction band and return to the ground state with decay process irradiation [32]. The PL spectra of BiOI/TNAs prepared through SILAR-ultrasonication assisted are shown in figure 15. From these spectra, the emission peaks of BiOI/TNAs and TNAs pristine peak at 538 nm are matched with the literature [29,49]. The PL intensity of TNAs pristine is higher than BiOI/TNAs heterojunction. The PL intensity shows the separation of charge carriers more efficiently by using an electrical field from the interface of the p-type of BiOI with a p-type semiconductor and an n-type TNAs semiconductor. The deposition of BiOI to TNAs has already improved charge carrier separation effectively [40]. The PL intensity shows the recombination of charge carrier photogeneration at a higher intensity [12,41]. The excellent PEC performance relates to the effectiveness of the photogenerated separation of electrons and holes [35]. The recombination rate of electrons and holes is highly affected when the BiOI is deposited on TNAs in large amounts [29].

The deposition of BiOI/TNAs has increased the effective charge separation, which is also in accordance with the results of Teng *et al*.'s [40] research. The lowest PL intensity resulted from TNAs B modified with BiOI at a concentration of 1 mM Bi and KI for 17 SILAR cycles (figure 15). A higher PL intensity indicates the recombination of many photogenerated charge carriers [41]. The presence of BiOI on TNAs can reduce the density of charge recombination centres for luminescence, characterized by a decreased PL intensity. BiOI nanosheets deposited on TNAs act as traps to capture photo-induced electrons from TNAs and thereby effectively inhibit the recombination of electron and hole pairs.

The PEC performance is closely related to separating photogenerated electron–hole pairs [35]. This PL spectrum shows that the peak emission power of pure TNA (bare TNAs) is higher than that of BiOI/TNAs. The PL spectra of BiOI/TNAs are lower than TNAs, which indicates the lower photosynthetic recombination efficiency resulting in better photocatalytic activity. Li *et al*. stated that when there are more layers deposited on the composite, it will also increase the recombination of photogenerated electrons [29]. The lower PL intensity shows the electron–hole pair recombination could be restrained [12]. The heterojunction formed at the interface of BiOI and TNAs improved the separation and migration of charge carrier photogeneration [12].

### 3.7. Characterization of photoelectrochemical properties by LSV and MPA

**Figure 16.**
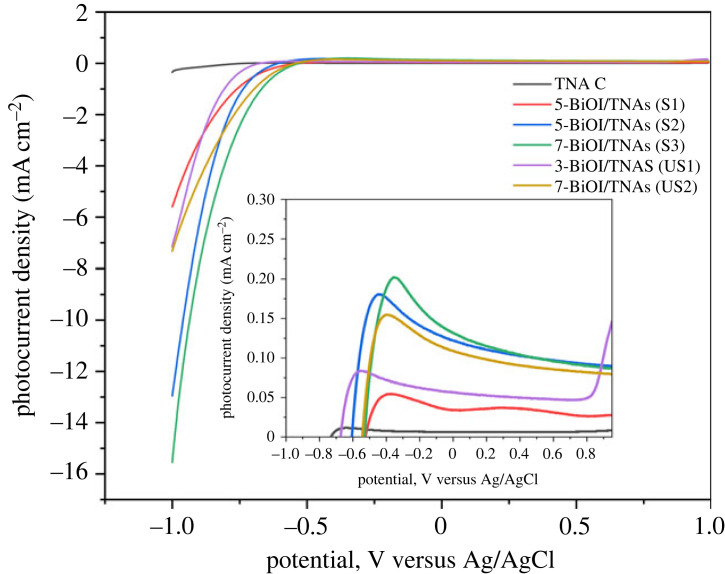
Current–voltage curve of TNAs 40 V 1 h, 50 V 1 h and BiOI/TNAs for 7 mM of Bi(NO_3_)_3_.5H_2_O illuminated by visible light of 13 W LED with a scan rate of 25 mV s^−1^ in 0.1 M Na_2_SO_4._

Figure 16 shows the LSV curve of TNAs bare and BiOI/TNAs at 7 mM precursors concentration by visible light illumination. From this LSV curve, TNAs have a photocurrent density of approximately 0.006 mA at 0 Volt vs Ag/AgCl. The photocurrent will be increased after being deposited with BiOI significantly. The maximum photocurrent density is influenced by cycles of deposition and precursor concentration. From this curve, for BiOI/TNAs of five cycles for 45 s per cycle, photocurrent density was 0.033 mA. The photocurrent density of BiOI/TNAs increases when the deposition time is 60 s. The highest photocurrent density resulted from the BiOI/TNAs with seven cycles, i.e. 0.130 mA. Otherwise, the photocurrent density is decreased for cycles of seven cycles for 90 s per cycle. The decreasing photocurrent is due to the overloading of BiOI on TNAs surface and inhibiting electron injection, and electron and hole are easier to recombine in the BiOI layer. The photocurrent onset of TNAs bare occurred at −0.72 V, and −0.51, −0.52, −0.54, −0.60 and −0.67 V for 5-BiOI/TNAs (SILAR-ultrasonication) 45 s, 5-BiOI/TNAs (SILAR-without ultrasonication) 60 s, 7-BiOI/TNAs (SILAR-without ultrasonication) 60 s, 3-BiOI/TNAs (SILAR-ultrasonication) 90 s and 7-BiOI/TNAs (SILAR-ultrasonication) 90 s, respectively. From this, the photocurrent onset of BiOI/TNAs showed better separation of electrons and holes and also the accumulation of electrons for BiOI/TNAs prepared by SILAR-assisted ultrasonication compared with SILAR without ultrasonication.

The modification of BiOI on TNAs increased the photocurrent density at the first step. As confirmed by SEM results, the photocurrent density is decreased due to the large particle size, and crystallite growth will form self-assembly. SILAR method with ultrasonication assisted has advantages to photocurrent density. Preparation of BiOI/TNAs heterojunction can increase homogeneity and faster crystallite growth. These results are confirmed with the XRD and UV-Vis DRS results.

**Figure 17.**
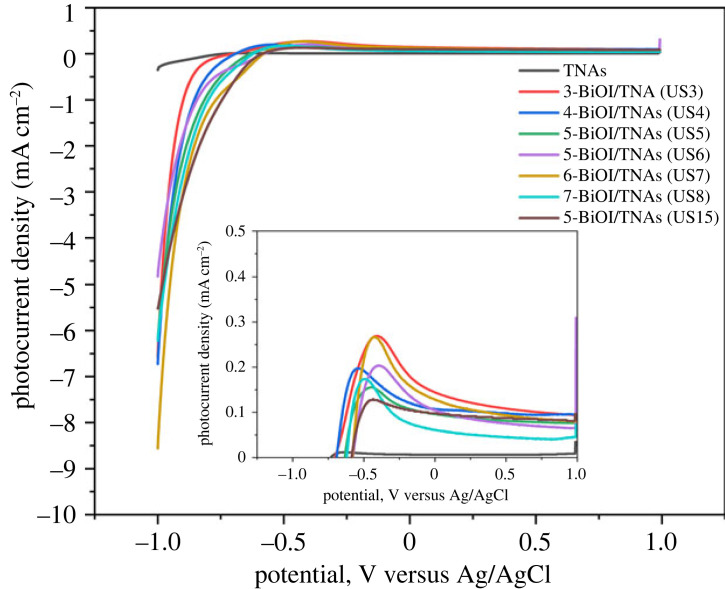
Current–voltage curve of TNAs (40 V 1 h, 50 V 1 h) and BiOI/TNAs illuminated with Visible light of 13 W LED with a scan rate of 25 mV s^−1^ in 0.1 M Na_2_SO_4_ with various concentrations of precursors.

The current–voltage curve of BiOI/TNAs prepared with 3 mM of cationic precursor and 2 mM of anionic precursor, 2 mM of cationic precursor and 2 mM of anionic precursor, and 3 mM of cationic precursor and 3 mM of anionic precursor with 25 mV s^−1^ of scan rate in an electrolyte solution of 0.1 M of Na_2_SO_4_ shown in figure 17. From this curve, the optimum photocurrent was 0.143 mA at 0 V vs Ag/AgCl. The synthesis condition for optimum photocurrent was three cycles for 60 s per cycle. The lowest photocurrent density at seven cycles at 2 mM of cationic precursor and 2 mM anionic precursor is approximately 0.061 mA. The small particle size benefited the photocurrent event with a concentration lower than 7 mM.

**Figure 18.**
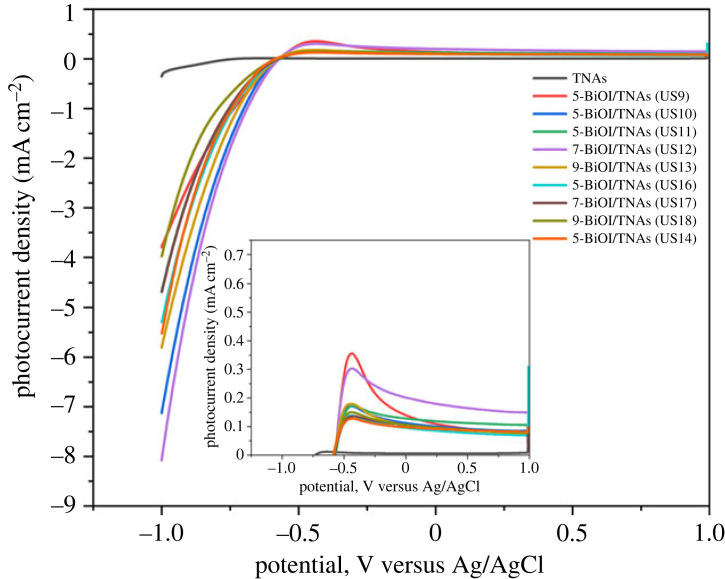
Current–voltage curve of BiOI/TNAs illuminated with visible light of 13 W LED for 5 mM of cationic precursor and 3 mM of anionic precursor; 3 mM of cationic precursor and 2 mM of anionic precursor; 2 mM of cationic precursor and 1 mM of anionic precursor; 3 mM of cationic precursor and 3 mM of anionic precursor with a scan rate of 25 mV s^−1.^

The LSV analysis of BiOI/TNAs with 5 mM of cationic precursor and 3 mM of anionic precursor; 3 mM of cationic precursor and 2 mM of anionic precursor; 2 mM of cationic precursor and 1 mM of anionic precursor; and 3 mM of cationic precursor and 3 mM of anionic precursor with the scan rate of 25 mV s^−1^ in an electrolyte solution of 0.1 M Na_2_SO_4_ for TNAs 40 V 60 min, 50 V 15 min (TNAs A) is shown in figure 18. The optimum photocurrent at 3 mM of cationic precursor and 2 mM of anionic precursor with seven cycles was 0.203 mA and for five cycles at 3 mM of cationic precursor and 2 mM of anionic approximately 0.130 mA.

**Figure 19.**
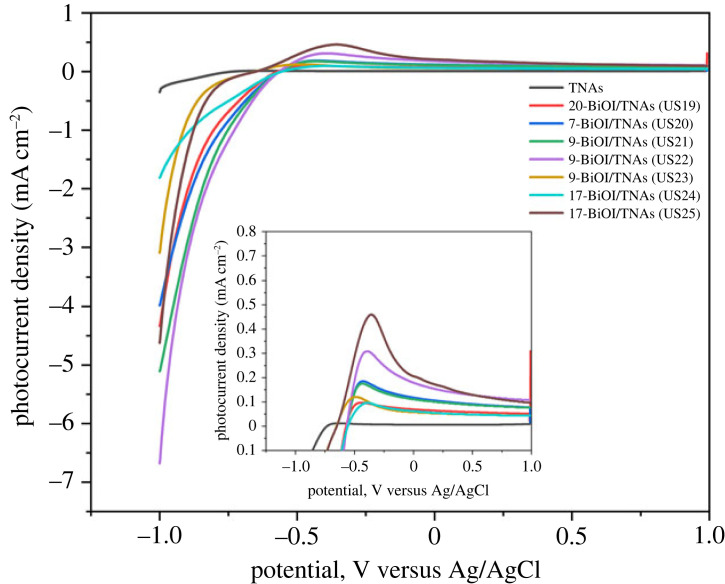
Current–voltage curve of TNAs and BiOI/TNAs illuminated with visible light of 13 W LED with 0.5 and 1 mM of cationic precursor with a scan rate of 25 mV s^−1^ in 0.1 M Na_2_SO_4._

The photocurrent density of BiOI/TNAs prepared with 0.5 mM of cationic precursor and 1 mM of anionic precursor with a 25 mV s^−1^ scan rate for TNAs A, B and C are depicted in figure 19. The optimum photocurrent density is reached for nine cycles at TNAs B (TNAs 40 V 60 min, 50 V 30 min). Decreasing the precursor concentration will increase the photocurrent density and be influenced by the tube morphology of TNAs. When the tube length of TNAs is shorter, the wall tube will be thicker, so if illuminated, many electrons and holes are produced. However, the tube length of TNAs must be sufficient for visible light absorption [50].

From this curve, all the samples show the increase of photocurrent with visible light illumination with repeating. The BiOI/TNAs photocurrent is higher than TNAs bare. The increasing photocurrent is due to the p-n junction of BiOI and TiO_2_, blocked by electron recombination [21]. The BiOI has a layered structure with [Bi_2_O_2_]^2+^ inserted along I directions on the atomic double plate (001). The internal static electric field between [Bi_2_O_2_]^2+^ and the anionic layer I was beneficial for effectively separating the photogeneration carrier [51]. The optimum photocurrent was obtained 5-BiOI/TNAs 3 mM cationic precursor and 2 mM anionic precursor. The higher photocurrent indicated a more effective separation of electrons and holes in the material [52].

**Figure 20.**
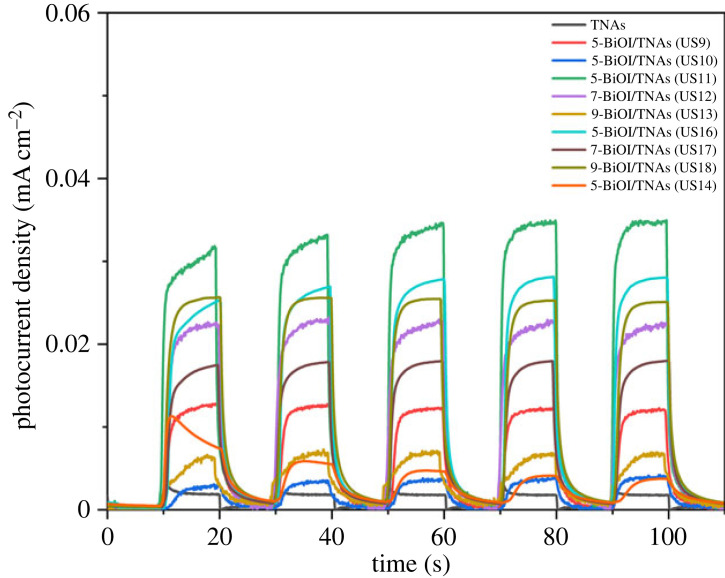
I–t curve of BiOI/TNAs illuminated with visible light of 13 W LED with 5 mM of cationic precursor and 3 mM of anionic precursor; 3 mM of cationic precursor and 2 mM of anionic precursor; 2 mM of cationic precursor and 1 mM of anionic precursor; and 3 mM of cationic precursor and 3 mM of anionic precursor.

The transient photocurrent (I-t) can provide information about electron transfer [2] in BiOI/TNAs. The transient photocurrent is recoded to TNAs bare and BiOI/TNAs with chopped visible light illumination using MPA with visible lamp 13 W LED at 0 V vs Ag/AgCl. I-t curves of BiOI/TNAs are shown in figure 20 with various concentrations of precursors on TNAs A (40 V for 60 min in the first step and 50 V for 15 min in the second step). Figure 20 displays the photocurrent transient for TNAs bare was 2 µA in the NaSO_4_ 0.1 M electrolyte solution. After deposition with BiOI semiconductor, the photocurrent density was higher than TNAs bare, which indicates better separation ability of photo-induced electron–hole pair [3,53]. The photocurrent density at various concentrations of 5 and 3 mM for cationic and anionic precursors is approximately 2.7 µA for TNAs (40 V for 45 min and 50 V for 15 min). When the concentration of cationic and anionic precursors was 3 and 2 mM, respectively, the photocurrent increased to 6.8 µA. The highest photocurrent density of BiOI/TNAs produced from the BiOI with cationic and anionic precursors was 3 and 2 mM for five cycles of 35.1 µA.

The increase in photocurrent is caused by forming a p-n junction between BiOI and TiO_2,_ which blocks electron recombination [21]. This BiOI has a layered structure with [Bi_2_O_2_]^2+^ inserted by double plates of I atoms along the (001) direction. The internal static electric field between the [Bi_2_O_2_]^2+^ and the anionic I layer is beneficial for effectively separating the photogenerated carriers. A higher photocurrent indicates a more effective separation of electrons and holes in the material [52]. The highest photocurrent was detected in BiOI/TNAs electrodes prepared at 3 mM Bi precursor concentrations and 2 mM KI for nine cycles, indicating a significantly increased electron density in these electrodes. The more electrons that are isolated, the more efficient the separation of the photogenerated carriers will be [12]. Therefore, the coupling of TNAs with BiOI resulted in more isolated electrons.

**Figure 21.**
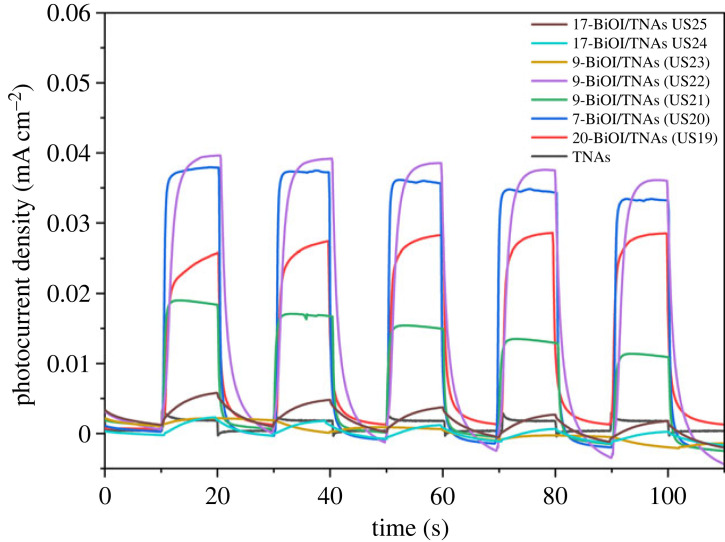
I–t curve of BiOI/TNAs illuminated with visible light of 13 W LED of BiOI/TNAs with a concentration of 0.5 and 1 mM of cationic precursor.

Figure 21 shows I-t curves of BiOI/TNAs at 0.5 and 1 mM of the precursors with various cycles SILAR on TNAs A, TNAs B and TNAs C. The optimum photocurrent of BiOI/TNAs prepared by 1 mM of cationic precursor and 1 mM of anionic precursor for nine cycles on TNAs B (TNAs 40 V 60 min, 50 V 30 min) was approximately 36.7 µA, which indicates that the electron density in BiOI/TNAs is increased and more isolated electrons [12]. This photocurrent is higher without bias potential (0 V vs Ag/AgCl). The 7-BiOI/TNAs 1 mM of cationic precursor and 1 mM of anionic precursor on TNAs A showed a photocurrent transient of 33.04 µA. The higher photocurrent of BiOI/TNAs B is due to the strong absorption of visible light caused by the BiOI nanoflake shape. The recombination level is lower from photo-induced internal electrostatic fields intersection of BiOI/TNAs heterojunction. The photocurrent density is also influenced by tube morphology, i.e. the diameter and length of the tube. If it is too long, injecting visible light is complicated, so recombination occurs quickly. If the tube length is too small, the surface area will be smaller, so the response to visible light is also tiny. This photocurrent value is higher than that of researchers Dai *et al*., where Dai *et al*. used an additional potential bias of 0.5 V vs Ag/AgCl [21]. The 7-BiOI/TNAs 1 mM Bi 1 mM KI TNAs C sample produced a photocurrent transient of around 33.04 µA. The increase in photocurrent is due to the attachment of BiOI to TNAs. The photoconductivity of TNAs is higher due to the separation and fast movement of pairs of electrons and holes [54]

Therefore, the optimum conditions for forming p-n heterojunctions between BiOI and TNAs were obtained at 9-BiOI/TNAs 1 mM Bi and 1 mM KI TNAs B (40 V 1 h, 50 V 30 min). According to the SEM results, the tube length through the cross-section test was around 7.00 ± 0.21 µm. The formation of a p-n heterojunction between BiOI/TNAs on TNAs 40 V 1 h, 50 V 1 h (TNAs C) results in a low photocurrent, which is also influenced by the thickness of the TNAs layer, which is around 12.53 ± 1.73 µm.

The transient photocurrent response can also indicate the photocatalytic activity of the electrode [4,55]. This photocurrent curve shows the anodic current surge at the start of irradiation for all samples (figure 21). After the lamp is turned off, the resulting current drops to zero. The initial spike in the photocurrent is caused by the generation and separation of photo-induced electron–hole pairs at the semiconductor and electrolyte interface. The holes will move on the surface of the semiconductor and then be captured by the reduced species in the electrolyte. At the same time, electrons are transferred along the TiO_2_ nanotube wall and the back contact of the Ti substrate. This decrease in photocurrent to constant shows that a few holes can reach the semiconductor surface. When the light is turned off, the photocurrent quickly drops to zero due to the rapid recombination of electron and hole pairs.

This photocurrent curve shows that the anodic current of BiOI/TNAs was almost stable for all variations as long as visible light was irradiated. When the visible light is on, the photocurrent increases sharply. It is owing to the generation and being moved apart of electron–hole pairs photo-induced at the interface of semiconductor and electrolyte. The holes move the surface of the semiconductor and catch reduced species in the electrolyte solution. Meanwhile, the electrons were transferred along the TNAs wall and the back contact of the Ti substrate. The descent of photocurrent to constant indicates that a small number of holes can achieve the surface of a semiconductor. When the visible light source is pressed in the off position, this causes the photocurrent quickly drops drastically to zero due to the rapid recombination of charge pairs (e^−^ and h^+^ pairs). The measurement of transient photocurrent by the MPA technique shows that the photocurrent generated for all BiOI/TNAs samples is higher than that of bare TNAs.

The MPA measurement shows that the resulting photocurrent for all BiOI/TNAs samples is higher than bare TNAs. A combination of parameters determines the increase in BiOI/TNAs photocurrent. First, the photocurrent is determined by the photogeneration efficiency of hole transfer in the semiconductor/electrolyte and electron diffusion in the back contact. Second, the p-n junction can reduce the recombination of photogenerated electrons and holes by having an internal electrostatic field within the junction. The presence of an external electrostatic field will increase the transfer and separation of photogenerated electrons and holes in BiOI/TNAs, thereby increasing the photocurrent density [21].

### 3.8. Hydrogen evolution in salty water using BiOI/TNAs photoanode

The DSSC device has been successfully fabricated using the Anode N719 Dyes/TNAs 40 V 1 h, 50 V 30 min. The efficiency of the DSSC is around 5.23%. The DSSC device will be used for DSSC-PEC tandem systems for hydrogen evolution in salty water.

**Figure 22.**
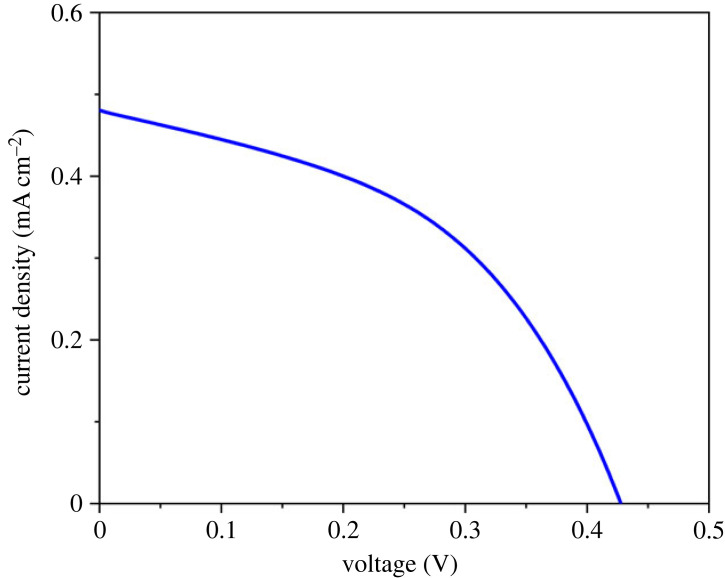
I–V curve of the solar device fabricated using N719 dyes/TNAs.

The current density–voltage (I–V) curve of DSSC fabricated by N719 dyes/TNAs is depicted in figure 22 with the illumination of a 19 W LED lamp using the LSV method with 0–1.0 V of potential range. The power conversion efficiency of DSSC was calculated using the formula in equation (3.4). Based on this equation, the power conversion efficiency of DSSC reached 5.23% with the open circuit (*V*_oc_)= 0.407, the short circuit current (*J*_sc_) = 0.483, the maximum current (*J*_max_), the maximum voltage (*V*_max_) and the fill factor (FF) = 45% has been shown in table 2.

3.4 $$\mathrm{FF}=\frac{\left(J_{\max}xV_{\max}\right)}{\left(J_{\mathrm{sc}}\;x\;V_{\mathrm{oc}}\right)}$$

**Table 2.** Parameter calculated from I–V measurement of DSSC fabricated.

*V*_oc_ (V)	*J*_sc_ (mA cm^−2^)	*V*_max_ (V)	*J*_max_ (mA cm^−2^)	*P*_max_ (mW cm^−2^)	*P*_in_ (mW cm^−2^)	FF (%)	*η* (%)
0.407	0.483	0.29	0.325	0.09425	1.8	45	5.23

Hydrogen production in salty water is carried out using a DSSC PEC tandem cell. BiOI/TNAs were used as a visible-illuminated anode. The Pt/TNAs compartment is used as a cathode, which acts as a catalytic zone. The hydrogen gas was collected with a gas bag sample and continued with the measurement using GC. The formula for calculating the efficiency of solar to hydrogen (STH) can be seen in equation (3.5).

**Figure 23.**
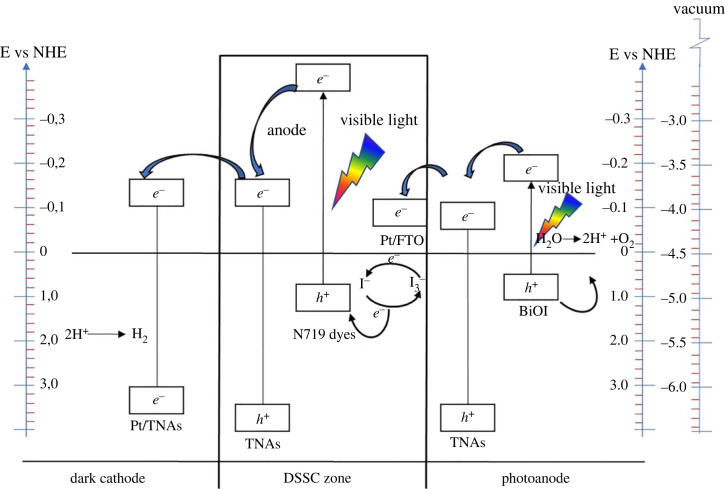
Energy schematic illustration of hydrogen evolution.

3.5 $$\eta =\frac{[{\mathrm{rH}}_{2}]\Delta G}{PA},$$

where *η* is efficiency of H_2_, [rH_2_] is the rate of hydrogen formation (mmol s^−1^), *ΔG* is the Gibbs free energy of hydrogen formation (237 kJ mol^−1^), *P* is the lamp power (mW cm^−2^) and *A* is area (cm^2^). The solar to hydrogen (STH) obtained 1.34% with the illumination of a 150 W metal halide lamp for 5 h. The STH of the photoanode is related to light harvesting, separation and transfer of photogenerated charge carriers [35]. The reaction mechanism of hydrogen evolution is shown in figure 23.

The hydrogen evolution was performed using a tandem DSSC-PEC system by illumination with a 150 W metal halide lamp. When absorbed energy equals or exceeds the band gap energy of the materials so that electrons are excited from the valence band (VB) to the conduction band (CB) and leave a hole (h^+^) in VB [56], the reaction can be seen in equation (3.6) [26].

3.6 $$\mathrm{BiOI}/\mathrm{TNAs}+\mathrm{hv}\to e^{-}(\mathrm{CB})+h^{+}(\mathrm{VB})$$

At the interface between BiOI and TNAs, charge transfer occurs with the reaction shown in equations (3.7) and (3.8). Red and *ox* are electrolyte conditions that experience reduction and oxidation.

3.7 BiOI (e+h) +TNAs→BiOI (h) +TNAs (e)

3.8 BiOI+Red→BiOI+ox

In the DSSC section, which is illuminated with visible light, the light will be absorbed by N719 dyes, and if the absorbed light equals or exceeds the band gap energy of N719 dyes, there will be excitation of electrons from the highest occupied molecular orbital (HOMO) of the dyes to the lowest unoccupied molecular orbital (LUMO) of the dyes. The reaction can be seen in reaction equation (3.9).

3.9 N719dyes+hv→N719dyes(h+e)

The generated electrons will be injected into the CB TNAs. The reaction that occurs at this time will leave holes in the VB N719 dyes. The reaction can be seen in reaction equation (3.10).

3.10 $${\mathrm{TiO}}_{2}+N719\mathrm{dyes}(\;h^{+}+e)\;\to {\mathrm{TiO}}_{2}(e)+N719\mathrm{dyes}(h^{+})$$

Electrons from the DSSC electrolyte will fill the remaining holes, resulting in a reduction process as in reaction equation (3.11).

3.11 $$S(h)+I_{3}^{-}\to S+I_{3}^{-}$$

In the DSSC, regeneration occurs, which results in the reduction of I_3_^−^ to I^−^ so that the N719 dyes return to their basic state. The reaction is shown in equation (3.12).

3.12 $$I_{3}^{-}+2e^{-}\to 3I^{-}$$

The electrons received by TiO_2_ above will then flow to the PEC cathode (Pt/TNAs), and in the presence of H^+^ ions and electrons transferred from TNAs, a reduction of H^+^ to H_2_ occurs. The reaction is as in equation (3.13).

3.13 $$\mathrm{Pt}/\mathrm{TNAs}(e^{-})+2H^{+}\to H_{2}$$

## 4. Conclusion

The morphology of BiOI/TNAs synthesized by the SILAR-ultrasonication assisted was nanoplates, nanosheets/nanoflakes. The BiOI were deposited not only on the surface of TNAs but also some flake of BiOI implants into the inner tube of TNAs. The orientation of BiOI was perpendicular to the TNAs matrix with varying concentrations of cationic and anionic precursors. The band gap energy of BiOI/TNAs is narrow, and the morphology of BiOI in the nanoplates form. Meanwhile, the more significant band gap will be in a nanoflake shape. SILAR cycles and SILAR time per cycle affect the morphology and the sheet size of BiOI produced on TNAs. The photoelectrochemical performance of BiOI modified on TNAs reveals the optimum photocurrent in the visible light for BiOI/TNAs of 1 mM of cationic and anionic precursors nine SILAR cycles with the TNAs that anodized at 40 V for 1 h in the first step and 50 V for 30 min in the second step anodization. The solar to hydrogen conversion efficiency (STH) of tandem DSSC-PEC reaches 1.34% in salty water.

## Ethics

This work does not contain any studies carried out on human participants or animals.

## Data Availability

Experimental data can be accessed from the Dryad Digital Repository: https://doi.org/10.5061/dryad.1vhhmgqx6 [57]. The data are provided in electronic supplementary material [58].

## References

[1] Koo MS, Cho K, Yoon J, Choi W. 2017 Photoelectrochemical degradation of organic compounds coupled with molecular hydrogen generation using electrochromic TiO_2_ nanotube arrays. Environ. Sci. Technol. **51**, 6590-6598. (10.1021/acs.est.7b00774) 28445067

[2] Wang P, Li H, Cao Y, Yu H. 2021 Carboxyl-functionalized graphene for highly efficient H_2_-evolution activity of TiO_2_ photocatalyst. Acta Phys. – Chim. Sin. **37**, 2008047.

[3] Li H, Sun B, Gao T, Li H, Ren Y, Zhou G. 2022 Ti_3_C_2_ MXene co-catalyst assembled with mesoporous TiO_2_ for boosting photocatalytic activity of methyl orange degradation and hydrogen production. Chinese J. Catal. **43**, 461-471. (10.1016/S1872-2067(21)63915-3)

[4] Mei Z, Wang G, Yan S, Wang J. 2021 Rapid microwave-assisted synthesis of 2D/1D ZnIn_2_S_4_/TiO_2_ S-scheme heterojunction for catalyzing photocatalytic hydrogen evolution. Acta Phys. – Chim. Sin. **37**, 1-11.

[5] Mokhtar SM, Ahmad MK, Soon CF, Nafarizal N, Faridah AB, Suriani AB, Mamat MH, Shimomura M, Murakami K. 2018 Fabrication and characterization of rutile-phased titanium dioxide (TiO_2_) nanorods array with various reaction times using one step hydrothermal method. Optik (Stuttg) **154**, 510-515. (10.1016/j.ijleo.2017.10.091)

[6] Kim JH, Hwang SM, Hwang I, Han J, Kim JH, Jo YH, Seo K, Kim Y, Lee JS. 2019 Seawater-mediated solar-to-sodium conversion by bismuth vanadate photoanode- photovoltaic tandem cell: solar rechargeable seawater battery. Iscience **19**, 232-243. (10.1016/j.isci.2019.07.024) 31382186 PMC6698286

[7] Jeong I, Park YH, Bae S, Park M, Jeong H, Lee P, Ko MJ. 2017 Solution-processed ultrathin TiO_2_ compact layer hybridized with mesoporous TiO_2_ for high-performance perovskite solar cells. ACS Appl. Mater. Interfaces. **9**, 36 865-36 874. (10.1021/acsami.7b11901) 28992419

[8] Liu J, Yao M, Shen L. 2019 Third generation photovoltaic cells based on photonic crystals. J. Mater. Chem. C **7**, 3121-3145. (10.1039/C8TC05461D)

[9] Ge M et al. 2017 A review of TiO_2_ nanostructured catalysts for sustainable H_2_ generation. Int. J. Hydrogen Energy. **42**, 8418-8449. (10.1016/j.ijhydene.2016.12.052)

[10] Qu Y, Zhu S, Dong X, Huang H, Qi M. 2022 Nitrogen-doped TiO_2_ nanotube anode enabling improvement of electronic conductivity for fast and long-term sodium storage. J. Alloys Compd. **889**(d), 161612.

[11] Szkoda M, Siuzdak K, Lisowska-Oleksiak A. 2016 Non-metal doped TiO_2_ nanotube arrays for high efficiency photocatalytic decomposition of organic species in water. Phys. E Low-Dimens. Syst Nanostructures **84**, 141-145. (10.1016/j.physe.2016.06.004)

[12] Li J, Wu C, Li J, Dong B, Zhao L, Wang S. 2022 1D/2D TiO_2_/ZnIn_2_S_4_ S-scheme heterojunction photocatalyst for efficient hydrogen evolution. Chin. J. Catal. **43**, 339-349. (10.1016/S1872-2067(21)63875-5)

[13] AlAqad KM, Kandiel TA, Basheer C. 2022 Synergy between in-situ immobilized MoS_2_ nanosheets and TiO_2_ nanotubes for efficient electrocatalytic hydrogen evolution. Int. J. Hydrogen Energy. **47**, 2366-2377. (10.1016/j.ijhydene.2021.10.159)

[14] Trang TNQ, Tu LTN, Man TV, Mathesh M, Nam ND, Thu VTH. 2019 A high-efficiency photoelectrochemistry of Cu_2_O/TiO_2_ nanotubes based composite for hydrogen evolution under sunlight. Comp. B: Eng. **174**, 106969. (10.1016/j.compositesb.2019.106969)

[15] Wang X, Guan Z-C, Jin P, Tang Y-Y, Song G-L, Liu G-K, Du R-G. 2019 Facile fabrication of BiVO_4_ modified TiO_2_ nanotube film photoanode and its photocathodic protection effect on stainless steel. Corros. Sci. **157**, 247-255. (10.1016/j.corsci.2019.05.034)

[16] Yu C, Zhang Z, Dong Z, Xiong Y, Wang Y, Liu Y, Cao X, Dong W, Liu YL M. 2021 Fabrication of heterostructured CdS/TiO_2_ nanotube arrays composites for photoreduction of U(VI) under visible light. J. Solid State Chem. **298**, 122053. (10.1016/j.jssc.2021.122053)

[17] Huang Y, Li H, Balogun MS, Liu W, Tong Y, Lu X, Ji H. 2014 Oxygen vacancy induced bismuth oxyiodide with remarkably increased visible-light absorption and superior photocatalytic performance. ACS Appl. Mater. Interfaces **6**, 22 920-22 927. (10.1021/am507641k) 25437430

[18] Yang J, Wang X, Lv X, Xu X, Mi Y, Zhao J. 2014 Preparation and photocatalytic activity of BiOX-TiO_2_ composite films (X = Cl, Br, I). Ceram. Int. **40**, 8607-8611. (10.1016/j.ceramint.2014.01.077)

[19] Jiang D, Chen L, Zhu J, Chen M, Shi W, Xie J. 2013 Novel p–n heterojunction photocatalyst constructed by porous graphite-like C_3_N_4_ and nanostructured BiOI: facile synthesis and enhanced photocatalytic activity. J. Chem. Soc. Dalt. Trans. **42**, 15 726-15 734. (10.1039/c3dt52008k) 24051513

[20] Li B, Chen X, Zhang T, Jiang S, Zhang G, Wu W, Ma X. 2018 Photocatalytic selective hydroxylation of phenol to dihydroxybenzene by BiOI/TiO_2_ pn heterojunction photocatalysts for enhanced photocatalytic activity. Appl. Surf. Sci. **439**, 1047-1056. (10.1016/j.apsusc.2017.12.220)

[21] Dai G, Yu J, Liu G. 2011 Synthesis and enhanced visible-light photoelectrocatalytic activity of p-n junction BiOI/ TiO_2_ nanotube arrays. J. Phys. Chem. C **115**, 7339-7346. (10.1021/jp200788n)

[22] Liu J, Ruan L, Adeloju SB, Wu Y. 2014 BiOI/TiO_2_ nanotube arrays, a unique flake-tube structured p–n junction with remarkable visible-light photoelectrocatalytic performance and stability. J. Chem. Soc. Dalt. Trans. **43**, 1706-1715. (10.1039/C3DT52394B) 24225559

[23] Ramakrishnan VM, Pitchaiya S, Muthukumarasamy N, Kvamme K, Rajesh G, Agilan S, Pugazhendhi A, Velauthapillai D. 2020 Performance of TiO_2_ nanoparticles synthesized by microwave and solvothermal methods as photoanode in dye-sensitized solar cells (DSSC). Int. J. Hydrogen Energy **45**, 27 036-27 046. (10.1016/j.ijhydene.2020.07.018)

[24] Gu P, Yang D, Zhu X, Sun H, Wangyang P, Li J, Tian H. 2017 Influence of electrolyte proportion on the performance of dye-sensitized solar cells. AIP Adv. **7**, 105219. (10.1063/1.5000564)

[25] An'Nur FK, Wihelmina B V, Gunlazuardi J, Wibowo R. 2020 Tandem system of dyes sensitized solar cell-photo electro chemical (DSSC-PEC) employing TiO_2_ nanotube/BiOBr as dark cathode for nitrogen fixation. In AIP Conf Proc., pp. 2243.

[26] Surahman H. 2017 Pengembangan Sel Fotoelektrokimia Menggunakan Elektroda TiO_2_ Nanotube Arrays Tersensitasi CdS Nanopartikel Untuk Produksi Hidrogen. In *Disertasi*.

[27] Wang K, Jia F, Zheng Z, Zhang L. 2010 Crossed BiOI flake array solar cells. Electrochem. Commun. **12**, 1764-1767. (10.1016/j.elecom.2010.10.017)

[28] Hou D, Hu X, Hu P, Zhang W, Zhang M, Huang Y. 2013 Bi_4_Ti_3_O_12_ nanofibers/BiOI nanosheets p-n junction: facile synthesis and enhanced visible-light photocatalytic activity. Nanoscale **5**, 9764-9772. (10.1039/c3nr02458j) 23963436

[29] Li M, Li L, Li B, Zhai L, Wang B. 2021 TiO_2_ nanotube arrays decorated with BiOBr nanosheets by the SILAR method for photoelectrochemical sensing of H_2_O_2_. Anal. Methods **13**, 1803-1809. (10.1039/D1AY00021G) 33885637

[30] Zhao WW, Liu Z, Shan S, Zhang WW, Wang J, Ma ZY, Xu JJ, Chen HY. 2014 Bismuthoxyiodide nanoflakes/titania nanotubes arrayed pn heterojunction and its application for photoelectrochemical bioanalysis. Sci. Rep. **4**, 1-6. 10.1038/srep04426 PMC3961734 24651880

[31] Xie YL, Guo LF, Ben CJ. 2022 Fabrication of BiOI nanoflowers decorated TiO_2_ nanotube arrays on porous titanium with enhanced photocatalytic performance for rhodamine B degradation. Int. J. Electrochem. Sci. **17**, 1-12.

[32] Yan T, Zhang X, Liu H, Jin Z. 2022 CeO_2_ particles anchored to Ni_2_P nanoplate for efficient photocatalytic hydrogen evolution. Jiegou Huaxue **41**, 2201047-53.

[33] Zhang X, Yang H, Zhang B, Shen Y, Wang M. 2016 BiOI-TiO_2_ nanocomposites for photoelectrochemical water splitting. Adv. Mater. Interfaces **3**, 3-7. (10.1002/admi.201500273)

[34] Mabuti LA, Manding IKS, Mercado CC. 2018 Photovoltaic and photocatalytic properties of bismuth oxyiodide-graphene nanocomposites. RSC Adv. **8**, 42 254-42 261. (10.1039/C8RA07360K) PMC9092079 35558407

[35] Li F, Dong B, Feng S. 2019 Bi shell-BiOI core microspheres modified TiO_2_ nanotube arrays photoanode: improved effect of Bi shell on photoelectrochemical hydrogen evolution in seawater. Int. J. Hydrogen Energy **44**, 29 986-29 999. (10.1016/j.ijhydene.2019.09.210)

[36] Jin YH, Li CM, Zhang YF. 2020 Preparation and visible-light driven photocatalytic activity of the rGO/TiO_2_/BiOI heterostructure for methyl orange degradation. Xinxing Tan Cailiao/New Carbon Mater. **35**, 394-400. (10.1016/S1872-5805(20)60496-6)

[37] Fan L et al. 2021 Visible-light-driven photoelectrochemical sensing platform based on BiOI nanoflowers/TiO_2_ nanotubes for detection of atrazine in environmental samples. J. Hazard. Mater. **409**, 124894. (10.1016/j.jhazmat.2020.124894) 33412470

[38] Zhang D. 2014 Heterostructural BiOI/TiO_2_ composite with highly enhanced visible light photocatalytic performance. Russ. J. Phys. Chem. A **88**, 2476-2485. (10.1134/S0036024414130044)

[39] Pishkar N, Ghoranneviss M, Ghorannevis Z, Akbari H. 2018 Physics study of the highly ordered TiO_2_ nanotubes physical properties prepared with two-step anodization. Results Phys. **9**, 1246-1249.

[40] Teng Q, Zhou X, Jin B, Luo J, Xu X, Guan H, Wang W, Yang F. 2016 Synthesis and enhanced photocatalytic activity of a BiOI/TiO_2_ nanobelt array for methyl orange degradation under visible light irradiation. RSC Adv. **6**, 36 881-36 887. (10.1039/C6RA01707J)

[41] Li S, Cai M, Liu Y, Zhang J, Wang C, Zang S, Li Y, Zhang P, Li X. 2022 *In situ* construction of a C_3_N_5_ nanosheet/Bi_2_WO_6_ nanodot S-scheme heterojunction with enhanced structural defects for the efficient photocatalytic removal of tetracycline and Cr(vi). Inorg. Chem. Front. **9**, 2479-2497. (10.1039/D2QI00317A)

[42] Costa LN, Nobre FX, Lobo AO, de Matos JME. 2021 Photodegradation of ciprofloxacin using Z-scheme TiO_2_/SnO_2_ nanostructures as photocatalyst. Environ. Nanotechnol. Monit. Manag. **16**, 100466.

[43] Cheng Y, Chen J, Wang P, Liu W, Che H, Gao X, Liu B, Ao Y. 2022 Interfacial engineering boosting the piezocatalytic performance of Z-scheme heterojunction for carbamazepine degradation: mechanism, degradation pathway and DFT calculation. Appl. Catal. B Environ. **317**, 121793. (10.1016/j.apcatb.2022.121793)

[44] Mehta M, Chandrabose G, Krishnamurthy S, Avasthi DK, Chowdhury S. 2022 Improved photoelectrochemical properties of TiO_2_-graphene nanocomposites: effect of defect induced visible light absorption and graphene conducting channel for carrier transport. Appl. Surf. Sci. Adv. **11**, 100274. (10.1016/j.apsadv.2022.100274)

[45] Sarkar A, Khan GG. 2019 The formation and detection techniques of oxygen vacancies in titanium oxide-based nanostructures. Nanoscale **11**, 3414-3444. (10.1039/C8NR09666J) 30734804

[46] Putri AA, Kato S, Kishi N, Soga T. 2019 Angle dependence of synthesized BiOI prepared by dip coating and its effect on the photovoltaic performance. Jpn J. Appl. Phys. **58**(SA), SAAD09. (10.7567/1347-4065/aaeb3b)

[47] Park Y, Na Y, Pradhan D, Min BK, Sohn Y. 2014 Adsorption and UV/visible photocatalytic performance of BiOI for methyl orange, rhodamine B and methylene blue: Ag and Ti-loading effects. CrystEngComm **16**, 3155-3167. (10.1039/C3CE42654H)

[48] Putri AA, Kato S, Kishi N, Soga T. 2019 Relevance of precursor molarity in the prepared bismuth oxyiodide films by successive ionic layer adsorption and reaction for solar cell application. J. Sci. Adv. Mater. Devices **4**, 116-124. (10.1016/j.jsamd.2019.01.007)

[49] Tian R, Liu D, Wang J, Zhou J, Nie E, Piao X, Sun Z. 2018 Three-dimensional BiOI/TiO_2_ heterostructures with photocatalytic activity under visible light irradiation. J. Porous Mater. **25**, 1805-1812. (10.1007/s10934-018-0594-3)

[50] Marien CBD, Cottineau T, Robert D, Drogui P. 2016 TiO2 Nanotube arrays: influence of tube length on the photocatalytic degradation of Paraquat. Appl. Catal. B Environ. **194**, 1-6. (10.1016/j.apcatb.2016.04.040)

[51] Ren X, Yao J, Cai L, Li J, Cao X, Zhang Y, Wang B, Wei Y. 2019 Band gap engineering of BiOI: via oxygen vacancies induced by graphene for improved photocatalysis. New J. Chem. **43**, 1523-1530. (10.1039/C8NJ05538F)

[52] Dai K, Lu L, Liang C, Zhu G, Liu Q, Geng L, He J. 2015 A high efficient graphitic-C_3_N_4_/BiOI/graphene oxide ternary nanocomposite heterostructured photocatalyst with graphene oxide as electron transport buffer material. Dalt. Trans. **44**, 7903-7910. (10.1039/C5DT00475F) 25823711

[53] Gao R, He H, Bai J, Hao L, Shen R, Zhang P, Li Y, Li X. 2022 Pyrene-benzothiadiazole-based polymer/CdS 2D/2D organic/inorganic hybrid S-scheme heterojunction for efficient photocatalytic H_2_ evolution. Chin. J. Struct. Chem. **4**, 31-45.

[54] Tayyab M, Liu Y, Min S, Irfan RM, Zhu Q, Zhou L, Lei J, Zhang J. 2022 Simultaneous hydrogen production with the selective oxidation of benzyl alcohol to benzaldehyde by a noble-metal-free photocatalyst VC/CdS nanowires. Chin. J. Catal. **43**, 1165-1175. (10.1016/S1872-2067(21)63997-9)

[55] Huang J, Li C, Hu X, Fan J, Zhao B, Liu E. 2022 K_2_HPO_4_-mediated photocatalytic H_2_ production over NiCoP/RP heterojunction. Jiegou Huaxue **41**, 2206062-8.

[56] Samsudin MFR, Sufian S, Mohamed NM, Bashiri R, Wolfe F, Ramli RM. 2018 Enhancement of hydrogen production over screen-printed TiO_2_/BiVO_4_ thin film in the photoelectrochemical cells. Mater. Lett. **211**, 13-16. (10.1016/j.matlet.2017.09.013)

[57] Kasuma Warda Ningsih S, Wibowo R, Gunlazuardi J. 2023 Data from: photoelectrochemical performance of BiOI/TiO_2_ nanotube arrays (TNAs) p-n heterojunction synthesized by SILAR-ultrasonication-assisted methods. Dryad Digital Repository. (10.5061/dryad.1vhhmgqx6) PMC10300691 37388319

[58] Kasuma Warda Ningsih S, Wibowo R, Gunlazuardi J. 2023 Photoelectrochemical performance of BiOI/TiO_2_ nanotube arrays (TNAs) p-n heterojunction synthesized by SILAR-ultrasonication-assisted methods. Figshare. (10.6084/m9.figshare.c.6472356) PMC10300691 37388319

